# Transcriptomic Analysis of Soil-Grown *Arabidopsis thaliana* Roots and Shoots in Response to a Drought Stress

**DOI:** 10.3389/fpls.2016.00180

**Published:** 2016-02-23

**Authors:** Sultana Rasheed, Khurram Bashir, Akihiro Matsui, Maho Tanaka, Motoaki Seki

**Affiliations:** ^1^Plant Genomic Network Research Team, RIKEN Center for Sustainable Resource SciencesYokohama, Japan; ^2^Kihara Institute for Biological Research, Yokohama City UniversityYokohama, Japan; ^3^CREST, Japan Science and Technology AgencySaitama, Japan

**Keywords:** abiotic stresses, abscisic acid, *Arabidopsis thaliana*, drought, microarray, transcription factors

## Abstract

Drought stress has a negative impact on crop yield. Thus, understanding the molecular mechanisms responsible for plant drought stress tolerance is essential for improving this beneficial trait in crops. In the current study, a transcriptional analysis was conducted of gene regulatory networks in roots of soil-grown *Arabidopsis* plants in response to a drought stress treatment. A microarray analysis of drought-stressed roots and shoots was performed at 0, 1, 3, 5, 7, and 9 days. Results indicated that the expression of many drought stress-responsive genes and abscisic acid biosynthesis-related genes was differentially regulated in roots and shoots from days 3 to 9. The expression of cellular and metabolic process-related genes was up-regulated at an earlier time-point in roots than in shoots. In this regard, the expression of genes involved in oxidative signaling, chromatin structure, and cell wall modification also increased significantly in roots compared to shoots. Moreover, the increased expression of genes involved in the transport of amino acids and other solutes; including malate, iron, and sulfur, was observed in roots during the early time points following the initiation of the drought stress. These data suggest that plants may utilize these signaling channels and metabolic adjustments as adaptive responses in the early stages of a drought stress. Collectively, the results of the present study increases our understanding of the differences pertaining to the molecular mechanisms occurring in roots vs. shoots in response to a drought stress. Furthermore, these findings also aid in the selection of novel genes and promoters that can be used to potentially produce crop plants with increased drought tolerance.

## Introduction

Adverse environmental factors, such as drought stress, severely limit agricultural production and reduce the yield and quality of crop plants. Water scarcity is predicted to increase as an outcome of climate change, and thus poses a serious challenge to agricultural production worldwide. Understanding the molecular response of plants to a drought stress and utilizing this knowledge for developing different molecular approaches to ameliorate the harmful effects of water deficit has always been an important objective for molecular breeders (Xiong et al., 2002; Umezawa et al., 2006; Yamaguchi-Shinozaki and Shinozaki, 2006; Seki et al., 2007; Shinozaki and Yamaguchi-Shinozaki, 2007; Hirayama and Shinozaki, 2010).

Plants sense changes in the environment and modify cellular physiology in a complex, integrated manner by upregulating the expression of various combinations of regulatory and functional genes. Despite a comprehensive knowledge of mechanisms governing cellular responses, our understanding of the early events in the perception of stress signals is relatively poor (Urao et al., 1999; Wohlbach et al., 2008). Drought stress triggers significant molecular and physiological changes in plants, such as adjustments of metabolism and osmotic potential, and reducing leaf turgor pressure, which lead to a reduction or cessation of growth (Tardieu et al., 2014). Although water deficiency inhibits plant growth at whole plant level, roots can grow under low water potentials that completely inhibit stem and leaf growth (Spollen and Sharp, 1991; Spollen et al., 1993; Chazen and Neumann, 1994; Wu and Cosgrove, 2000; Sharp et al., 2004). Since increasing root surface area facilitates water absorption, it is plausible that differences between roots and shoots may have evolved in response to water scarcity as an adaptation strategy to dry conditions (Wu and Cosgrove, 2000; Sharp et al., 2004). Cellular and molecular responses underlying adaptation to environmental stresses have been extensively studied and are governed by changes in gene expression (Matsui et al., 2008; Liu et al., 2014). Changes in the expression of a large number of genes belonging to diverse functional groups, such as transcription factors, protein kinases, and phosphatases, all contribute to the signal transduction that occurs in plants in response to and adaptation to a drought stress (Kreps et al., 2002; Seki et al., 2002, 2007; Xiong et al., 2002; Shinozaki et al., 2003; Matsui et al., 2008; Hirayama and Shinozaki, 2010).

Drought stress response has been extensively studied in *Arabidopsis* and the subject has been comprehensively reviewed (Iuchi et al., 2001; Xiong et al., 2002; Seki et al., 2007; Shinozaki and Yamaguchi-Shinozaki, 2007; Matsui et al., 2008; Harb et al., 2010; Osakabe et al., 2014). Stress-responsive genes are comprised of enzymes regulating osmotic pressure, aquaporins, detoxifying enzymes, late embryogenesis abundant proteins, reactive oxygen species scavengers and chaperones that protect the integrity of cell membranes and ensure ion transport/balances. Additionally, functionally diverse transcription factors and protein kinases, that regulate gene expression and signal transduction, are also an integral component of the drought stress response (Wei et al., 2009). The molecular response of plants to drought stress has been categorized into abscisic acid (ABA) dependent and ABA independent pathways (Yamaguchi-Shinozaki and Shinozaki, 2006). ABA biosynthesis, transport and accumulation all increase in response to a water deficit. The increased ABA content in leaves triggers stomata closure, ultimately decreasing the rate of gas exchange, respiration, and photosynthetic activity (Yamaguchi-Shinozaki and Shinozaki, 2006). An increase in the endogenous ABA content also induces the expression of a number of stress-related genes in plants (Yamaguchi-Shinozaki and Shinozaki, 2006). Briefly, the ABA signaling pathway affects plant adaptation to stress by regulating the internal water status in plants (Osakabe et al., 2014). The ABA independent pathway is mainly regulated by dehydration-responsive element/C-repeat (DRE/CRT) and DRE-/CRT-binding protein 2 (DREB2) transcription factors (Yamaguchi-Shinozaki and Shinozaki, 2006).

As previously mentioned, the root system is the first to perceive drought stress signals. Therefore, root development is significantly affected by water availability in the soil. Most studies in *Arabidopsis*, however, have explored transcriptomic changes in whole plants by only investigating shoots on soil-grown plants or air-dried roots. Therefore, at the present time, the drought response of roots in soil-grown plants remains largely unknown. To fill this gap, changes in the expression of genes in roots and shoots of soil-grown plants in response to a progressive drought stress were characterized and compared by sampling plants at 0, 1, 3, 5, 7, and 9 days of a drought stress. This provided the opportunity to dissect the molecular response of shoots vs. roots to a drought stress. The objective of the study was to obtain information that could be used to develop new strategies for developing drought tolerant plants.

## Materials and methods

### Plant material and growth conditions

Seeds of *Arabidopsis thaliana* (Col-0 ecotype) were grown on MS medium at 22°C under 16-h-light/8-h-dark (40–80 μmol photons m^−2^ s^−1^) for 9 days. Plantlets were then transferred to ceramics granular soil (size 2.5L, Sakatanotane, Japan) and grown for 8 days at 22°C (16 h light/8 h dark cycle, 60 μmol m^−2^ s^−1^ photon flux density). The drought treatment was then commenced by removing excess water from the trays and ceasing any subsequent watering. Roots and shoots were harvested separately at 0, 1, 3, 5, 7, and 9 days after the onset of drought treatment. Plants were removed from soil and roots and shoots of 12 plants were harvested from 3 pots for each biological replication. All samples were collected at noon. After harvesting, samples were immediately placed in liquid nitrogen and stored at −80°C until RNA extraction.

### Microarray analysis

RNA was extracted from all biological replicates with the *mir*Vana™ miRNA Isolation Kit (Ambion, USA) according to the manufacturer's instructions. The microarray analyses were carried out as previously described (Nguyen et al., 2015). Briefly, fluorescent-labeled (Cy3) cRNAs were prepared from 400 ng total RNA from each sample using a Quick Amp labeling kit (Agilent Technologies) and subsequently hybridized to an Agilent *Arabidopsis* custom microarray (GPL19830). Three biological replicates were processed for each treatment, with the exception of roots 7 and 9 days as well as shoot 1 and 3 days, where four biological replications were processed, giving a total of 40 hybridizations. Arrays were scanned with a microarray scanner (G2505B, Agilent Technologies) and the R 2.12.1 software program (R Core Team). RMA normalization was performed for the obtained signals of the microarray probes using limma package (Ritchie et al., 2015). A student's *t*-test (*p*-value) was performed as a parametric test and the Benjamini and Hochberg False Discovery Rate (FDR; *q*-value) procedure was used to control the certainty level (Benjamini and Hochberg, 1995). Genes with at least a 2-fold change in expression and having a *q* < 0.1 were considered to be differentially expressed. The microarray data has been deposited to GenBank with accession number GSE76827.

### MapMan and gene ontology (GO) analysis

The average log_2_ value of all biological replicates was calculated separately for roots and shoots for individual annotations at each time point. Gene ontology analyses were carried out using the PANTHER (protein annotation through evolutionary relationship) classification system database maintained at http://pantherdb.org/ (Mi et al., 2013). The GO analyses were performed for molecular function, protein classification and pathway. To further validate the results, the normalized log_2_ values were then used to compare the transcriptomic changes using MapMan 3.6.0RC1(Thimm et al., 2004). PageMan analysis was also performed using MapMan 3.6.0RC1 which included a Wilcoxon test with BH correction (Thimm et al., 2004).

### Real time PCR

Real time PCR analysis was performed for *RD29A* (*AT5G52310*), *NCED2* (*AT4G18350*), *NCED3* (*AT3G14440*), and *GolS4* (*AT1G60470*) genes with standard curve method in order to confirm that plants were experiencing water stress and to confirm the results obtained by microarray analysis. cDNA for each sample was synthesized from 200ng RNA using QuantiTect Rev. Transcription Kit according to the manufacturer's protocol (QIAGEN, USA). For *NCED2* the forward and reverse primers were 5′-CGCCGGTTTGGTT TACTTTA-3′ and 5′-GCGTGAAGCTCC TTCGTAAC-3′ respectively. Forward and reverse primers used for *NCED3* were 5′-ACTCATGCTATT CTACGCCAGAG-3′ and 5′-ACCAACGGTTT TTAAATCTCCAT-3′, respectively. For *RD29A* the forward and reverse primers were 5′-TGGATCTGAAGAA CGAATCTGATATC-3′ and 5′-GGTCTT CCCTTCGCCAGAA-3′, respectively. For *GolS4* the forward and reverse primers were 5′- TTGCCATGGCTTA TTACGTTC -3′ and 5′-AAACAGTCCATCACGGCATAG-3′, respectively. *Actin 2*, used as an internal control, was amplified using the forward and reverse primers 5′-TGAAGTGTGATGTGGATATCAGG-3′ and 5′-GATTTCTTTGCTCATACGGTCAG-3′, respectively.

## Results

### General transcriptional changes in roots and shoots during early drought stress

**Figure 1:**
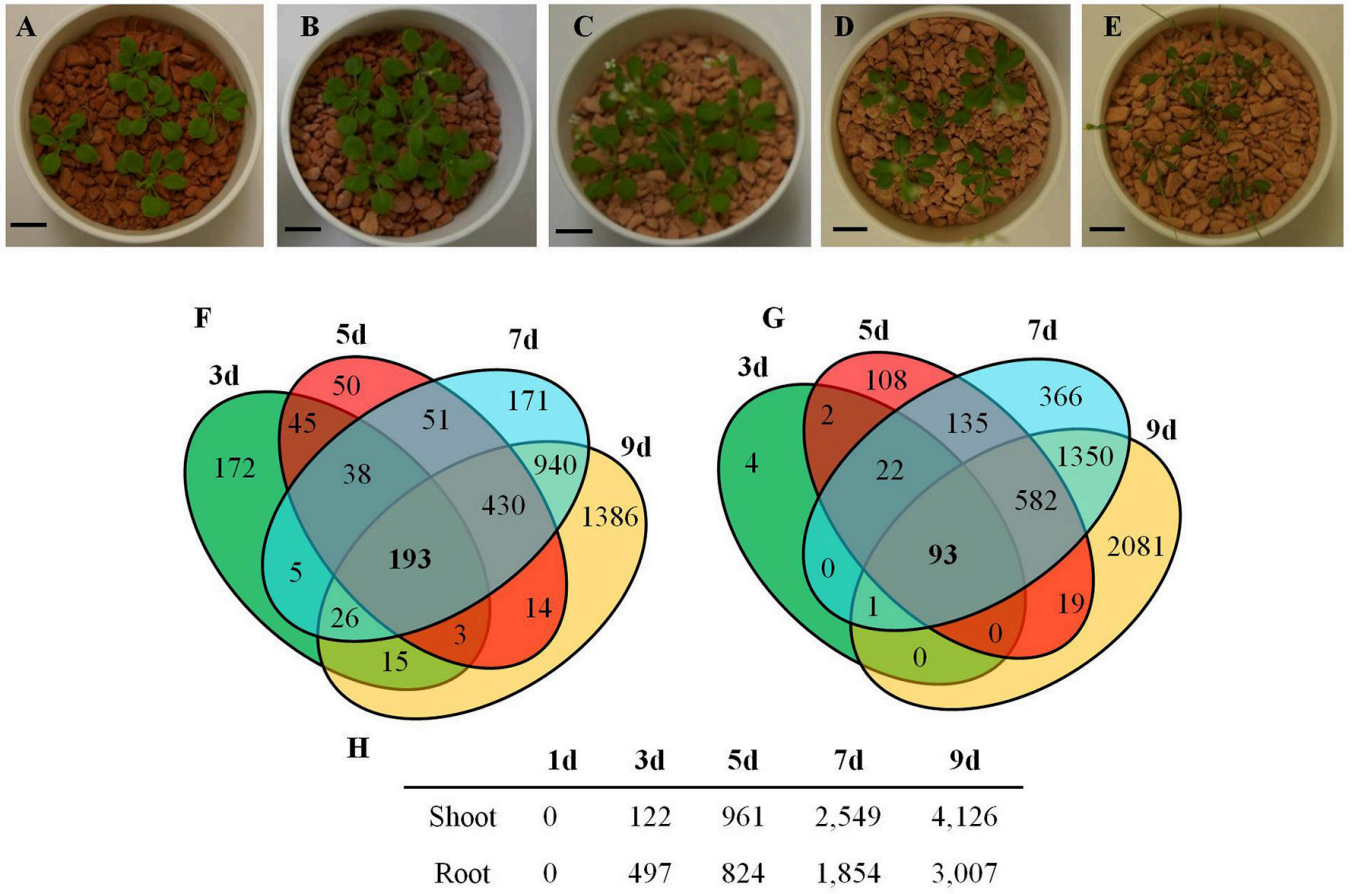
*****Arabidopsis*** genes up-regulated by a progressive drought stress. (A–E)** Plants grown in ceramics soil under drought conditions, **(A)** Day 0, **(B)** Day 3, **(C)** Day 5, **(D)** Day 7, and **(E)** Day 9 of the drought stress treatment. **(F)** Venn diagram of the number of genes up-regulated by drought stress in roots. **(G)** Venn diagram of the number of genes up-regulated by drought stress in shoots. **(H)** Number of genes up-regulated in roots and shoots during a progressive drought stress. **(A–E)** Scale bar = 1cm. **(F–H)** Genes with at least a 2-fold increase in expression and having a FDR < 0.1 were considered to be up-regulated.

**Figure 2:**
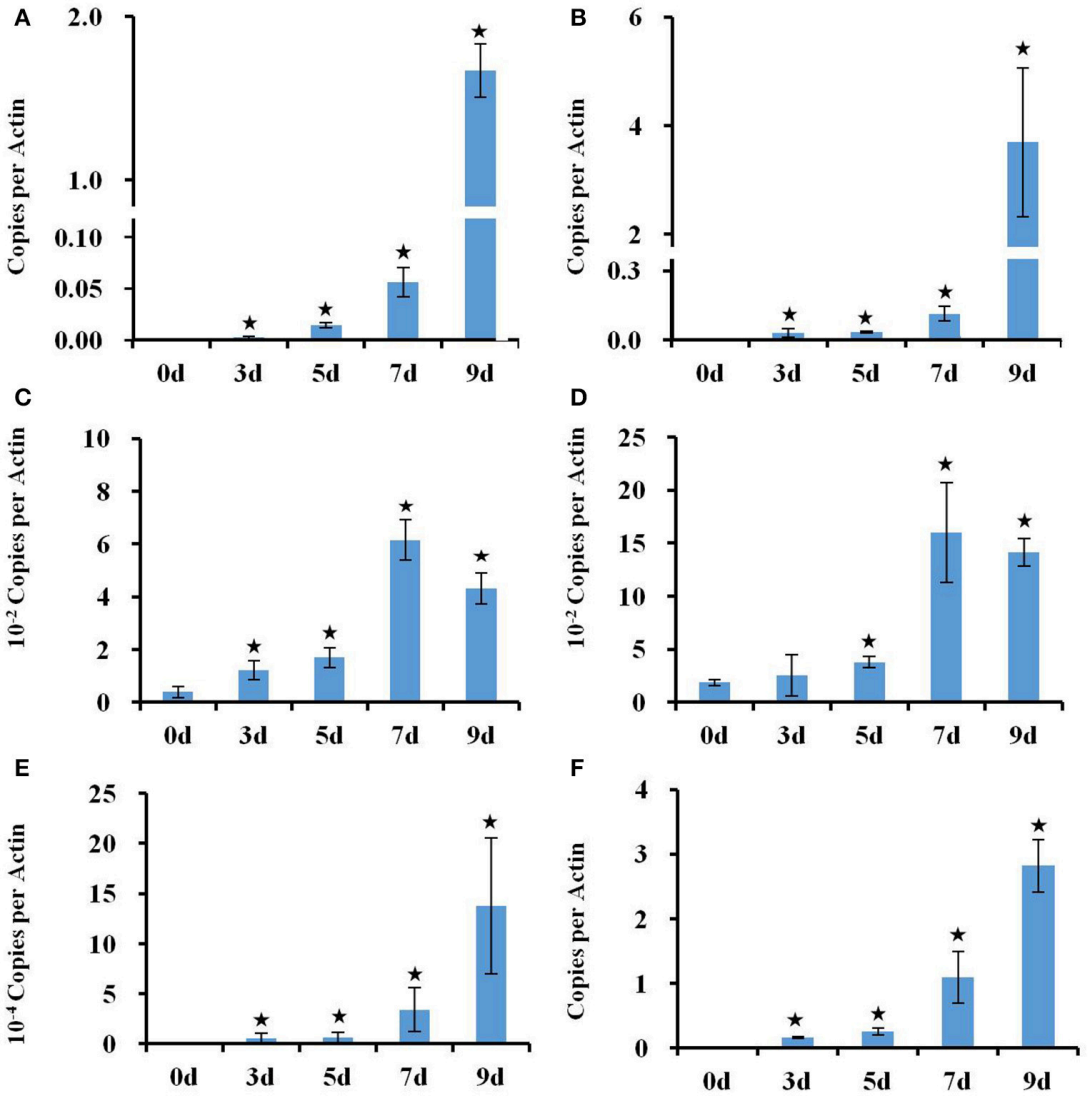
**Real time PCR analysis of the expression of ***RD29A, NCED3***, ***NCED2***, and ***GolS4*** genes in response to a progressive drought stress. (A,B)** Expression of *RD29A* gene in roots **(A)** and shoots **(B)**, **(C,D)** Expression of *NCED3* in roots **(C)** and shoots **(D)**. Expression of *NCED2* **(E)** and *GolS4* **(F)** in roots. *Actin 2* expression was used to normalize the expression of all genes. Column bars with an asterisk above are significantly different from Day 0 based on a *t*-test (*p* < 0.05).

The transcriptional changes in roots and shoots of soil grown plant subjected to progressive drought were analyzed. The water retention capacity of ceramics granular soil is poor due to its large pore size, thus the drought stress increased rapidly relative to normal soil, and all plants had died by day 10 of the drought stress (data not shown). The pots started to dry around day 5 but no morphological symptoms of drought stress were observed by that time (Figure S1). By day 9 the plants appeared wilted (Figures 1A–E), however, plants could recover if they were watered (data not shown). In roots and shoots, no genes were observed to be significantly up or down-regulated in either roots or shoots on day 1 of the drought treatment. At day 3 of the drought treatment, 497 genes were significantly up-regulated in roots (Figure 1 and Table S1), while 292 genes were significantly down-regulated (Figure S2 and Table S2). At 5, 7, and 9 days of the drought treatment, the number of up-regulated genes in roots was 824, 1,854, and 3,007 respectively (Figure 1). The number of down-regulated genes in roots at days 5, 7, and 9 of the drought treatment were 899, 2327, and 3742, respectively (Figure S2 and Table S2). In total, 3539 genes were up-regulated and 4154 genes were down-regulated in roots. Similar to roots, no genes were observed to be significantly up- or down-regulated in shoots on day 1 of the drought treatment. On day 3 of the drought treatment, 122 genes were significantly up-regulated in shoots (Figure 1 and Table S3), while 91 genes were significantly down-regulated (Figure S2 and Table S4). On days 5, 7, and 9 of the drought treatment, the number of up-regulated genes in shoots was 961, 2549, and 4126, respectively (Figure 1). On the other hand, the number of down-regulated genes was 528, 2442, and 4848, respectively (Figure S2 and Table S2). In total, 4763 genes were up-regulated and 5213 genes were down-regulated in shoots. The expression of 1906 genes was up-regulated in both roots and shoots (Table S5), while the expression of 2218 genes was down-regulated (Table S6) in both roots and shoots (Figure S2). To determine the reliability of results of the microarray analysis, the expression of four genes (*NCED2, NCED3, RD29A*, and *GolS4*) that were up-regulated by drought stress was examined by real time PCR. The results of the real time PCR analysis confirmed the results obtained using the microarray (Figure 2). Genes that were up-regulated in roots at least 4-fold on day 3 of the drought stress, as compared to 0 day, are listed in Table 1. These genes belong to diverse functional groups, including oxygenases, cytochrome P450 family proteins, Multidrug And Toxin Extrusion (MATE) efflux transporters, and *RD29A, RD29B* (*AT5G52300*), etc., and may play an important role in early drought response.

**Table 1:** *****Arabidopsis*** genes highly up-regulated in roots during the early stages of a progressive drought stress**.

**Gene**	**Name**	**Root**				**Shoot**			
		**3 days**	**5 days**	**7 days**	**9 days**	**3 days**	**5 days**	**7 days**	**9 days**
*At1g52820*	*2OG-Fe(II)-dependent oxygenase*	**7.1**	**5.5**	1.0	**0.4**	0.8	1.0	1.0	1.0
*At1g32350*	*AOX1*	**4.0**	0.9	1.9	**3.6**	0.9	0.8	1.0	**2.5**
*At4g35690*	*Protein of unknown function (DUF241)*	**4.3**	**6.9**	**9.8**	**8.7**	1.5	1.1	**3.1**	**8.1**
*At5g28510*	*BGLU24*	**6.0**	**18.2**	**47.5**	**37.7**	1.2	0.9	1.5	1.6
*At2g37870*	*Bifunctional inhibitor/lipid-transfer 2S albumin superfamily protein*	**16.9**	**110.3**	**195.5**	**227.1**	7.6	**48.2**	**183.7**	**229.6**
*At4g33550*	*Same as above*	**7.7**	**48.3**	**92.4**	**115.3**	3.0	**20.6**	**51.9**	**115.3**
*At5g52310*	*RD29A*	**6.7**	**26.5**	**41.1**	**75.1**	**4.6**	**4.7**	**17.8**	**24.3**
*At5g52300*	*RD29B*	**8.4**	**68.4**	**164.7**	**335.9**	**5.7**	**7.2**	**76.6**	**116.6**
*At4g23700*	*Cation/H^+^ exchanger 17*	**4.3**	**3.0**	**5.1**	**5.0**	0.5	**0.4**	**0.3**	1.7
*At3g13784*	*βFRUCT6/CWINV5*	**5.5**	**7.4**	**5.4**	**9.9**	1.5	1.5	**11.7**	**48.4**
*At2g43570*	*Chitinase, putative*	**4.6**	1.5	1.9	**2.6**	1.2	0.9	1.3	**5.8**
*At4g37220*	*Cold accl. protein /WCOR413 family*	**4.5**	**2.4**	**2.3**	**3.0**	1.1	1.4	**3.6**	**3.6**
*At1g73810*	*Core-2/I-branching beta-1,6-N-acetyl glucosaminyltransferase family protein*	**4.9**	**3.5**	**4.2**	**7.0**	1.5	1.5	1.3	**2.4**
*At5g50260*	*CEP1*	**7.9**	**5.2**	**2.8**	**4.2**	1.2	1.4	**8.7**	**13.7**
*At5g36130*	*CYP 450 superfamily protein*	**12.5**	**5.6**	1.1	**0.2**	0.9	1.0	1.0	1.0
*At5g47990*	*CYP705A5*	**4.3**	**4.1**	**2.5**	0.7	0.9	0.9	0.9	0.7
*At2g30750*	*CYP71A12*	**4.1**	1.5	0.5	**0.5**	0.7	0.5	**0.5**	0.5
*At2g34500*	*CYP710A1*	**4.4**	**2.3**	**2.1**	**2.3**	0.8	**0.5**	0.9	**12.9**
*At5g36140*	*CYP716A2*	**9.1**	**4.4**	1.1	**0.4**	1.3	1.1	1.1	1.2
*At5g66400*	*DI8/RAB18/RESPONSIVE to ABA 18*	**4.2**	**79.7**	**157.6**	**316.7**	3.4	**19.1**	**334.6**	**825.6**
*At3g21520*	*DUF679 domain membrane protein 1*	**4.3**	1.4	2.0	1.5	1.5	0.8	1.0	1.4
*At1g26390*	*FAD-binding Berberine family protein*	**12.9**	2.2	1.1	1.4	0.7	**0.2**	**0.1**	**0.1**
*At1g26410*	*FAD-binding Berberine family protein*	**6.8**	1.3	**0.4**	0.5	0.7	**0.4**	**0.3**	**0.3**
*At1g60470*	*Galactinol synthase 4/GolS4*	**4.9**	**10.3**	**28.4**	**21.6**	1.2	1.1	1.4	**3.5**
*At4g19810*	*Glycosyl hydrolase family protein*	**4.4**	**2.5**	1.5	**2.1**	1.1	0.5	0.6	**2.6**
*At5g59220*	*Highly ABA-induced PP2C1*	**7.8**	**25.0**	**86.5**	**101.0**	3.1	**5.9**	**74.3**	**110.8**
*At1g07430*	*Highly ABA-induced PP2C2*	**7.4**	**30.9**	**115.5**	**170.2**	2.9	**6.9**	**66.8**	**73.7**
*At2g29380*	*Highly ABA-induced PP2C3*	**8.3**	**37.3**	**116.1**	**156.9**	1.3	1.3	**12.6**	**36.4**
*At2g39050*	*EULS3*	**4.0**	**5.5**	**8.1**	**12.6**	1.7	1.8	**4.0**	**7.3**
*At1g18870*	*Isochorismate synthase 2*	**10.6**	**20.6**	**14.2**	**2.1**	**2.9**	**3.6**	**2.1**	0.7
*At5g06760*	*Late Embryogenesis Abundant (LEA) 4-5*	**12.3**	**59.9**	**199.0**	**400.6**	3.2	**15.4**	**426.2**	**759.0**
*At1g52690*	*LEA7 very specific to drought*	**16.9**	**144.3**	**274.4**	**562.6**	3.5	**72.4**	**2663.5**	**3664.4**
*At3g15670*	*LEA76*	**13.9**	**128.1**	**221.8**	**636.6**	1.3	1.4	**55.7**	**604.4**
*At5g01550*	*Lectin receptor kinase A4.2*	**5.2**	1.6	1.7	1.6	1.1	1.0	0.9	**3.1**
*At5g59310*	*Lipid transfer protein 4*	**15.6**	**385.9**	**371.5**	**680.2**	**20.7**	**288.9**	**950.2**	**1016.0**
*At5g28520*	*Mannose-binding lectin superfamily*	**7.7**	**31.1**	**65.3**	**22.9**	1.3	0.9	1.2	1.3
*At5g42600*	*Marneral synthase*	**11.7**	**17.3**	**17.2**	**6.3**	0.7	0.8	0.9	1.0
*At5g19700*	*MATE efflux transporter/ABS3L1*	**4.2**	1.9	1.0	1.0	1.1	1.3	1.3	1.2
*At2g16005*	*MD-2-related lipid recognition domain-containing protein*	**10.3**	**36.3**	**50.9**	**38.7**	1.0	0.9	0.8	1.4
*At1g73220*	*OCT1*	**4.5**	**5.0**	**2.5**	**2.8**	1.0	1.9	**2.8**	**2.2**
*At1g34510*	*Prx8*	**5.3**	**3.8**	**3.2**	1.3	1.0	1.1	1.0	1.0
*At5g04120*	*Phosphoglycerate mutase family protein*	**24.8**	**32.6**	**27.2**	**8.5**	0.9	0.9	0.9	1.1
*At1g70720*	*Invertase/pectin methylesterase inhibitor*	**4.2**	**6.6**	**5.2**	**2.2**	1.3	**4.9**	**12.0**	**5.9**
*At3g17130*	*Same as above*	**4.0**	**5.9**	**8.8**	**5.6**	1.8	1.5	**3.5**	1.5
*At1g31750*	*Proline-rich family protein*	**4.2**	**9.8**	**28.2**	**40.9**	1.7	**3.2**	**20.0**	**32.3**
*At3g28300*	*Protein of unknown function (DUF677)*	**4.2**	**7.8**	**11.3**	**8.5**	2.0	2.0	**2.1**	0.6
*At1g09310*	*Protein of unknown function, DUF538*	**4.3**	**20.5**	**23.0**	**24.0**	1.1	0.9	1.1	**0.4**
*At3g18250*	*Putative membrane lipoprotein*	**4.5**	0.9	0.6	0.9	0.8	0.7	**0.3**	**0.3**
*At5g36150*	*Putative pentacyclic triterpene synthase 3*	**9.5**	**5.7**	1.0	**0.4**	0.8	1.1	1.2	1.0
*At3g08860*	*PYRIMIDINE 4*	**5.6**	**4.8**	**2.4**	1.0	2.9	**6.2**	**7.4**	**2.7**
*At3g49580*	*Response to low sulfur 1*	**6.4**	**3.7**	**2.8**	0.9	1.8	**4.7**	**2.1**	**0.3**
*At5g38910*	*RmlC-like cupins superfamily protein*	**20.3**	3.2	1.2	1.8	1.1	0.8	0.8	0.9
*At4g25220*	*Root hair specific 15*	**4.8**	2.2	1.4	**0.4**	0.8	0.8	0.8	0.8
*At1g66700*	*SABATH methyltransferase PXMT1*	**4.9**	1.7	1.1	1.9	0.9	0.5	0.5	1.0
*At5g13170*	*SWEET15*	**5.3**	**30.6**	**73.8**	**160.2**	4.9	**22.9**	**108.0**	**193.9**
*At2g40250*	*SGNH hydrolase-type esterase*	**5.6**	**6.5**	1.9	0.7	0.9	0.8	0.6	0.5
*At5g25260*	*FLOT1A*	**4.5**	1.0	**0.5**	0.6	1.3	0.5	**0.3**	**0.3**
*At4g21650*	*Subtilase 3.31*	**5.1**	**17.2**	**23.8**	**5.9**	2.3	**6.9**	**10.4**	1.9
*At4g21640*	*Subtilase family protein*	**4.6**	**15.9**	**23.2**	**5.5**	**2.6**	**7.8**	**12.4**	**2.4**
*At4g21630*	*Subtilase family protein*	**4.9**	**15.7**	**24.1**	**5.6**	2.4	**7.3**	**11.8**	**2.3**
*At5g11110*	*Sucrose phosphate synthase 2F/SPSA2*	**5.8**	**15.5**	**32.6**	**64.4**	1.2	1.5	**5.5**	**10.6**
*At5g65990*	*Transmembrane amino acid transporter*	**6.7**	**13.5**	**24.6**	**20.0**	1.3	1.1	**2.3**	1.8
*At2g19410*	*U-box domain-containing protein kinase*	**5.1**	2.9	**2.6**	1.9	0.9	0.8	1.0	0.8
*At1g21240*	*Wall associated kinase 3/WAK3*	**4.6**	0.7	0.6	0.8	1.6	1.1	**0.3**	**0.4**
*At1g03790*	*Zinc finger C-x8-C-x5-C-x3-H type*	**4.0**	**28.5**	**69.0**	**230.0**	1.1	1.4	**8.5**	**138.0**

*Values shown in bold are significantly up or down-regulated (FDR < 0.1)*.

In general, the drought-inducible genes were up-regulated in roots at a very early stage of the drought stress treatment (days 3–5), while in shoot tissue this response was slightly delayed (days 5–7). For example, the expression of *protein phosphatase 2C1* (*PP2C1*; *AT5G59220*), *PP2C2* (*AT1G07430*), and *PP2C3* (*AT2G29380*) was significantly up-regulated in roots on days 3–9 of the drought stress. The expression of *PP2C1* and *PP2C2* was up-regulated in shoots on days 5–9, while the expression of *PP2C3* was up-regulated on days 7–9 of the drought stress (Table S1). The expression of *DREB2A* (*AT5G05410*) was up-regulated in roots from day 5 to 9, and in shoots from day 7 to 9. *DREB2B* (*AT3G11020*) was up-regulated in roots from day 7 to 9, and only at day 9 in shoots (Table S1).

**Figure 3:**
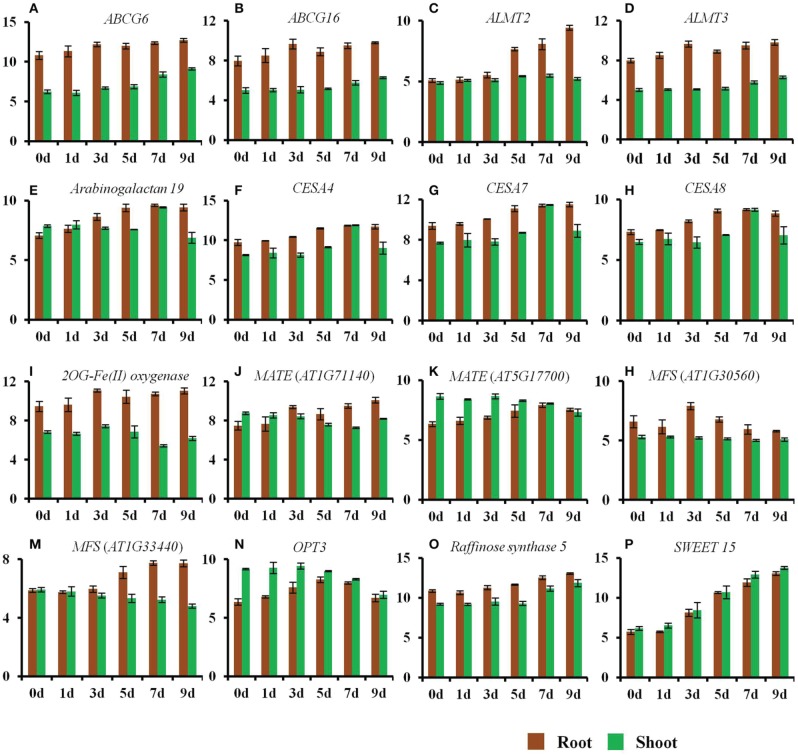
**Changes in the expression of genes by a progressive drought stress**. The normalized log_2_ values were used to plot the expression of genes regulated by a progressive drought stress. **(A)** *ABCG6*, **(B)** *ABCG16*, **(C)** *ALMT2*, **(D)** *ALMT3*, **(E)** *Arabinogalactan protein 19*, **(F)** *CESA4*, **(G)** *CESA7*, **(H)** *CESA8*, **(I)** *2OG-Fe(II) oxygenase (AT4G10500)*, **(J)** *MATE* transporter (*AT1G71140*), **(K)** *MATE* transporter (*AT5G17700*), **(L)** *MFS* transporter (*AT1G30560*), **(M)** *MFS* transporter (*AT1G33440*), **(N)** *OPT3*, **(O)** *Raffinose synthase 5*, **(P)** *SWEET 15*. Error bars represent standard deviation.

Our data revealed that 1633 genes were specifically up-regulated in roots (Figure S2) and was examined in relation to previously published tiling microarray results (Matsui et al., 2008). In comparison to the genes up-regulated in response to 2 or 10 h of drought stress identified in the tiling array, (Matsui et al., 2008), the current analysis identified 1353 new genes (83% of total genes specifically up-regulated in roots) that were specifically up-regulated in roots in response to drought stress (Table S1). The newly identified genes were members of diverse gene families such as major facilitator super family (MFS) transporters [*AT1G08900, AT1G30560, AT1G33440, AT1G72140, AT1G80530, AT2G26690, AT2G34355, AT3G20460, AT3G45680, AT3G47960, AT4G19450, STP8 (AT5G26250), AT5G27350*, and *AT5G62680*], MATE efflux transporters (*AT1G71140, AT5G17700, AT5G19700*, and *AT5G38030*), microRNA genes [*MIR156b* (*AT4G30972), MIR161* (*AT1G48267*), *MIR162b* (*AT5G2306*5), *MIR164* (*AT5G01747*), *MIR167c* (*AT3G04765*), *MIR168b* (*AT5G45307*), *MIR396a* (*AT2G10606*), *MIR402* (*AT1G77235*), *MIR777a* (*AT1G70645*), and *MIR848a* (*AT5G13887*)], various transcription factors (*MYB, NAC* domain, WRKY, etc.), ABA biosynthesis-related genes (*NCED5, NCED9*), pectin biosynthesis/modification-related genes, pre-tRNA genes, and various S-adenosyl-L-methionine (SAM) dependent transferases (Figure 3 and Table S1). In comparison to the tiling array conducted by Matsui et al. (2008), 1,724 additional genes were identified in the current study that were specifically down-regulated in roots (Table S2). Moreover, our data also revealed the differential regulation of several genes in roots vs. shoots (Tables S1, S3) that have been already reported to be involved in drought stress response (Huang et al., 2008; Matsui et al., 2008).

### Gene ontology (GO) and MapMan analysis

**Figure 4:**
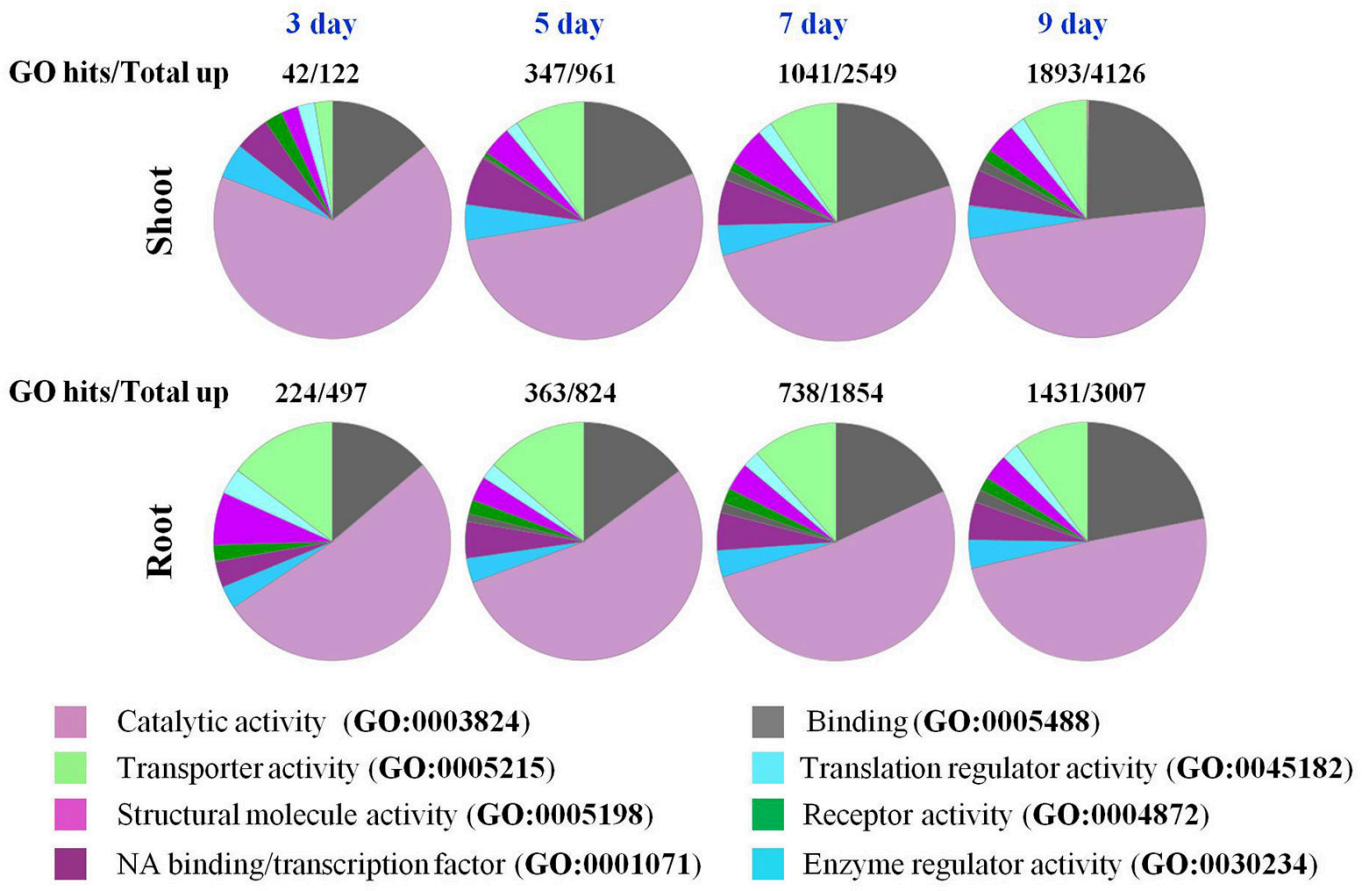
**GO enrichment analysis of genes in roots and shoots of ***Arabidopsis plants*** that respond to a progressive drought stress**. Go functional classification was performed using the panther classification system maintained at http://pantherdb.org/.

GO enrichment analysis revealed that the majority of the up-regulated genes in roots and shoots on day 3 of the drought treatment belonged to catalytic activity (GO:0003824; Figure 4), however, in roots a significant number of up-regulated genes were also identified as related to transport (GO:0005215) and structural molecular activity (GO:0005198). As the drought stress progressed, genes belonging to molecular binding (GO:0005488), transport (GO:0005215), and transcription factors (GO:0001071) were also up-regulated in shoots (Figure 4). At day 7 and 9 of the drought stress, the response of roots and shoots seemed very similar (Figure 4). The number of up-regulated genes in roots on day 3 of the drought stress was higher than the number of up-regulated genes in shoot tissue, while an opposite trend was observed on days 5–9 of the drought stress (Figure 1). GO enrichment analysis indicated that different transporters/transport related genes were significantly up-regulated in roots compared to shoots on day 3 of the drought stress (Figure 4). The number of up-regulated genes involved in structural molecule activity (GO:0005198) was also higher in roots compared to shoots (Figure 4). GO enrichment analysis for protein classification revealed that a number of calcium binding proteins (PC00060) were also up-regulated in roots on day 3 of drought stress (Figure S3). The most striking differences observed between roots and shoots were at day 3 of drought stress for pathway analysis. In shoots, genes involved in general transcription regulation (P00023) and transcription regulation by bZIP transcription factor (P00055) were recognized by GO analysis, while in roots genes belonging to 28 pathways were recognized (Figure S4). Majority of genes up regulated or down regulated both in roots and shoots were classified as engaged in metabolic or cellular process according to GO analysis (Figure S5).

The MapMan and GO analyses are comparable to each other, which serves as a justification for comparative analysis (Klie and Nikoloski, 2012). MapMan and PageMan analysis were done to validate the GO enrichment analysis and to categorize the genes in more detail. MapMan analysis indicated that a greater number of genes categorized as cell wall biosynthesis related genes, lipid metabolism related genes, and genes involved in secondary metabolism were also up-regulated in roots compared to shoots on day 3 of the drought stress (Figures S6, S7). MapMan analysis revealed that genes involved in photosynthesis/light reactions were significantly down-regulated in shoots starting at day 5 of the drought stress (Figures S6, S7). In shoots, genes involved in minor CHO metabolism and cell wall synthesis were up-regulated at days 7 and 9 of the drought stress, while almost all the genes involved in photosynthesis/light reactions were significantly down-regulated (Figure S7).

PageMan analysis of roots revealed that bins related to major carbohydrate (CHO) metabolism, cell wall synthesis, and DNA and chromatin structure were significantly up-regulated, while bins relating to amino acid metabolism, and nucleic acid metabolism were down-regulated (Figure S8). Bins related to mitochondrial electron transport (shoots), amino acid metabolism (roots and shoots), nucleotide metabolism (roots and shoots) were significantly down-regulated at an early stage of drought stress (from day 3), while bins related to development and RNA synthesis/transcription (shoot) became significantly up-regulated as the drought treatment progressed (Figure S8).

### Changes in the expression of cell wall and suberin synthesis genes

Roots rapidly sense changes in water potential and significantly alter roots architecture in an attempt to acquire more water in order to maintain a non-detrimental water potential. This is evident by the changes in the expression of genes belonging to cell wall, suberin, and lignin biosynthesis. Expression of ABC transporters involved in lignin transport also increased in roots (Table S1). The expression of cellulose synthase (CES) genes, *CESA4* (*AT5G44030*), *CESA7* (*AT5G17420*), and *CESA8* (*AT4G18780*) was significantly up-regulated in roots on days 5–9 of the drought stress (Figure 3). These genes have been reported to contribute to secondary cell wall synthesis (Carpita, 2011). In contrast, the expression of genes involved in primary cell wall synthesis (*CESA1*; *AT4G32410, CESA3*; *AT5G05170, CESA6*; *AT5G64740*) was not altered in roots. Moreover, the expression of *ABCG6* (*AT5G13580*) and *ABCG16* (*AT3G55090*), was significantly up-regulated in roots at a very early stage of the drought stress (Figure 3). These genes belong to a set of five *Arabidopsis* ABCG transporters that are required for synthesis of an effective suberin barrier in roots and seed coats (ABCG2; *AT2G37360*, ABCG6, and ABCG20; *AT3G53510*) and for synthesis of an intact pollen wall (ABCG1; *AT2G39350* and ABCG16) (Yadav et al., 2014). The expression of arabinogalactan protein 19 (*AT1G68725*) was also up-regulated in roots from day 3 to 9 of the drought stress (Figure 3). This gene contributes to plant growth, as mutants for this gene show reduced height, altered leaf shape and size, and lighter color (Yang et al., 2007).

### Regulation of osmoprotectant biosynthesis-related genes

The expression of genes involved in the biosynthesis of osmoprotectants changed significantly during the early stages of the drought stress, particularly in roots. Raffinose and galactinol are involved in tolerance to drought, high salinity, and cold stresses. Galactinol synthase (GolS) catalyzes the first step in the biosynthesis of raffinose (Taji et al., 2002). Seven GolS (GolS1-7) members have been reported in *Arabidopsis*. The expression of *GolS1* (*AT2G47180*) and *GolS2* (*AT1G56600*) is up-regulated by drought stress. Plants over expressing *GolS2* exhibit increased levels of endogenous galactinol and raffinose, and are tolerant to drought stress (Taji et al., 2002). The data in the current study indicate that the expression of *GolS1* was specifically up-regulated (Table S3), while that of *GolS3* (*AT1G09350*) was specifically down-regulated in shoots on day 7 and 9 of the drought stress (Table S4). The expression of *GolS2* was up-regulated in roots from days 5 to 9 of the drought stress, while in shoots it was up-regulated from day 3 to 9 (Table S1). The expression of *GolS4* was significantly up-regulated in roots from day 3 to 9, while in shoots it was up-regulated only on day 9 of the drought stress (Table 1). The expression of *raffinose synthase 5* (*RS5*; *AT5G40390*) was up-regulated in roots and shoots on days 7 and 9 of the drought stress (Figure 3). Changes in the expression of genes involved in proline synthesis were also observed. The expression of *P5CS1* (*AT2G39800*) was up-regulated in roots and shoots from day 5 to 9 (Table S1), while the expression of *P5CS2* (*AT3G55610*) was specifically up-regulated in shoots on day 7 and 9 (Table S3).

### Transcriptional changes in ABA and other hormone-related genes

A number of hormone-related genes were significantly up-regulated in roots and shoots (Table 2). Among genes in the ABA biosynthesis pathway, *NCED2* was up-regulated on day 3, while *CYP707A1* (*AT4G19230*), *NCED3*, and *NCED9* (*AT1G78390*) were up-regulated in roots from day 5 to 9 of the drought stress (Table 2). Thus, it is reasonable to conclude that ABA biosynthesis was up-regulated around day 5 of the drought stress. The up-regulation of *NCED*2 and *NCED3* occurred earlier in roots than in shoot tissue. In contrast, the expression of *AAO3* was specifically up-regulated in shoots. The expression of transcription factors involved in ABA response also changed differentially in roots and shoots. The expression of *AREB1/ABF2* (*AT1G45249*), *AREB2/ABF4* (*AT3G19290*), and *ABF3* (*AT4G34000*) was reported to be up-regulated in vegetative tissues in response to drought, high salinity, and ABA (Fujita et al., 2005). In the present study, the expression of *AREB1/ABF2* was up-regulated in roots from day 5 to 9 of the drought stress, and from day 7 to 9 in shoots. The expression of *AREB2/ABF4* was specifically up-regulated in shoots on day 7 and 9, while the expression of *ABF3* was up-regulated in roots on day 7. On the other hand, it was up-regulated from day 3 to 9 of the drought stress in shoots (Table 2 and Table S3).

**Table 2:** **Changes in expression of hormone related genes**.

**Gene**	**Name**	**Root**				**Shoot**			
		**3 days**	**5 days**	**7 days**	**9 days**	**3 days**	**5 days**	**7 days**	**9 days**
**ABA RELATED GENES**									
*At2g27150*	*AAO3*	0.82	0.97	1.02	1.74	1.13	1.21	**2.59**	**9.59**
*At5g67030*	*ABA1/ZEP*	1.19	1.65	1.87	**3.13**	1.23	1.24	1.04	**2.90**
*At1g52340*	*ABA2*	1.09	1.08	0.96	0.81	0.84	0.69	0.59	0.62
*At1g16540*	*ABA3*	*0.90*	0.86	1.43	**2.53**	0.98	1.10	1.66	**3.95**
*At4g19230*	*CYP707A1*	1.80	**2.54**	**6.61**	**16.05**	1.11	**2.15**	**3.56**	**12.73**
*At5g45340*	*CYP707A3*	0.36	0.31	**0.11**	**0.15**	0.84	1.64	**0.40**	0.60
*At4g18350*	*NCED2*	**2.67**	**13.86**	**25.85**	**22.63**	0.90	1.15	**3.62**	1.58
At3g14440	*NCED3*	6.37	**11.04**	**39.5**	**35.88**	1.78	11.04	**5.09**	**4.55**
*At4g19170*	*NCED4*	1.05	1.07	1.04	1.37	1.62	**2.19**	0.71	0.88
*At1g30100*	*NCED5*	1.69	1.48	**2.37**	1.84	1.25	1.26	1.30	0.69
*At3g24220*	*NCED6*	0.87	0.96	1.01	1.30	1.00	0.91	1.44	1.43
*At1g78390*	*NCED9*	1.82	**3.01**	**3.57**	**2.11**	1.01	1.13	1.68	1.98
*At1G52400*	*BG1*	2.29	**6.05**	**40.41**	**13.68**	1.66	**2.06**	**2.16**	**0.24**
*At5g06530*	*ABCG22*	**4.43**	**11.46**	**17.98**	**24.46**	1.25	1.14	1.58	0.89
*At1g45249*	*AREB1/ABF2*	1.88	**3.40**	**7.07**	**6.26**	1.30	1.84	**8.77**	**7.83**
*At3g19290*	*AREB2*	1.04	1.20	1.83	1.84	0.95	1.07	**2.53**	**2.13**
*At4g34000*	*ABF3*	1.57	1.52	**2.35**	1.94	**2.16**	**3.84**	**9.47**	**6.15**
*At1g71960*	*ABCG25*	1.73	**1.99**	**2.15**	1.77	1.27	1.65	**2.36**	**2.44**
*At1g15520*	*ABCG40*	1.84	0.83	**0.34**	**0.47**	0.71	**0.14**	**0.16**	**0.11**
*At1g69850*	*AIT1*	1.34	1.69	1.50	**2.09**	0.79	0.79	0.64	**0.47**
**AUXINS RELATED GENES**									
*At4g32540*	*YUCCA1*	1.24	**2.46**	**2.01**	**3.58**	0.84	0.89	0.69	0.68
*At5g11320*	*YUCCA4*	1.56	1.81	1.69	**3.15**	1.07	1.00	1.07	0.97
*At1g70560*	*TAA1/SAV3*	1.08	1.69	**2.36**	**4.73**	0.77	0.99	0.80	0.89
*At1g34060*	*TAR4*	1.23	1.01	0.81	0.87	**2.05**	**4.87**	**2.28**	1.83
*At5g28237*	*TSB2 homol*.	0.98	1.07	1.14	0.97	1.19	**2.36**	**3.16**	1.12
*At3g44300*	*NIT2*	1.63	1.29	**2.05**	**2.72**	1.05	0.57	0.81	**2.32**
*At2g30770*	*CYP71A13*	1.50	1.40	1.34	**2.93**	0.37	0.13	0.08	0.33
**CYTOKININ RELATED GENES**									
*At4g24650*	*IPT4*	0.94	**2.58**	1.11	0.98	1.05	1.02	0.91	0.97
*At2g28305*	*LOG1*	0.84	1.44	**5.53**	**7.51**	0.94	1.73	**4.31**	**3.80**
*At2g35990*	*LOG2*	**2.80**	**2.22**	1.50	0.78	1.12	1.11	1.06	1.07
*At3g53450*	*LOG4*	0.76	1.28	1.58	**3.06**	0.89	0.89	1.37	**2.88**
*At4g35190*	*LOG5*	**3.21**	**2.25**	**2.92**	**2.38**	1.20	**3.60**	**4.75**	**3.90**
*At5g56970*	*CKX3*	0.80	0.91	0.53	0.21	1.79	**2.15**	1.32	1.13
*At1g75450*	*CKX5*	1.71	**2.11**	**2.15**	1.60	1.01	1.15	1.11	0.92
*At5g05870*	*UGT76C1*	1.29	1.99	**3.52**	**3.74**	1.15	0.96	1.60	1.51
*At2g36750*	*UGT73C1*	1.00	1.30	1.35	**3.98**	1.34	1.05	**3.00**	**13.49**
*At2g36800*	*UGT73C5*	**3.19**	1.04	1.67	**2.53**	1.24	1.28	0.67	0.69
*At1g22400*	*UGT85A1*	1.08	1.07	0.92	0.77	0.52	0.36	0.72	**3.51**
*At4g22570*	*APT3*	0.80	1.23	1.67	0.88	1.30	1.93	**2.10**	0.47
**GIBBERELLINS(GA) RELATED GENES**									
*At1g79460*	*KS/GA2*	1.01	1.67	**2.47**	**3.17**	1.34	1.61	1.70	1.20
*At5g25900*	*KO/GA3*	0.65	0.74	0.98	1.08	1.08	1.18	1.39	**2.40**
*At5g51810*	*GA20ox2*	0.90	1.81	1.76	1.79	0.94	1.17	**2.86**	1.40
*At1g44090*	*GA20ox5*	1.35	1.53	1.59	**2.48**	0.81	0.87	1.00	1.17
*At1g02400*	*GA2ox6*	0.84	0.81	1.16	**2.34**	0.43	1.05	1.51	**5.07**
**ETHYLENE BIOSYNTHESIS PATHWAY GENES**									
*At1g01480*	*ACS2*	**2.03**	1.39	**2.97**	**7.93**	0.88	1.05	**7.92**	**41.88**
*At5g28360*	*ACS3*	1.04	0.93	1.06	1.03	1.16	1.14	**2.25**	1.12
*At4g26200*	*ACS7*	1.18	0.65	0.54	0.83	0.95	0.90	0.99	**2.26**
**REGULATION OF JASMONATE (JA) RELATED GENES**									
*At3g25760*	*AOC1*	1.22	0.83	0.76	0.71	1.13	**2.14**	0.63	0.21
*At4g16760*	*ACX1*	1.54	1.90	**3.43**	**3.89**	1.06	1.07	1.73	**3.70**
*At5g65110*	*ACX2*	1.41	1.22	1.78	**2.65**	1.02	0.83	1.54	**2.90**
*At3g51840*	*ACX4*	0.86	0.75	0.84	1.14	1.01	1.12	1.24	**2.36**
*At5g07010*	*ST2a*	1.16	1.53	**2.04**	**2.94**	0.64	0.54	1.24	**4.36**
*At3g25760*	*AOC1*	1.22	0.83	0.76	0.71	1.13	**2.14**	0.63	0.21

*Values shown in bold are significantly up or down-regulated (FDR < 0.1)*.

Similarly, the expression of transporters involved in ABA transport and ABA-induced stomatal closure also responded differently in roots and shoots. An increase in the expression of *ABCG25* (*AT1G71960*) was observed in roots on day 7, while the increase in shoots occurred on day 7–9 of the drought stress. The expression of *ABCG22* (*AT5G06530*) was specifically up-regulated in roots. The expression of *ABCG40* (*AT1G15520*) was significantly down-regulated in both roots and shoots in response to the drought stress. Down-regulation of *ABCG40* was observed in roots on days 7 and 9, and from day 5 to 9 in shoots (Table 2). The expression of the ABA transporter, *AIT1* (*AT1G69850*), was significantly up-regulated in roots on day 9 of the drought stress, while it was significantly down-regulated in shoots (Table 2).

The expression of auxin biosynthesis-related genes also displayed differential patterns of expression in roots vs. shoots (Table 2). The expression of *YUCCA1* (*AT4G32540*) and *tryptophan aminotransferase of Arabidopsis 1* (*TAA1/SAV3; AT1G70560*) was specifically up-regulated in roots. The expression of *NITrilase 2* (*NIT2; AT3G44300*) was up-regulated in roots on days 7 and 9 and only on day 9 in shoots (Table 2). The expression of *tryptophan aminotransferase related 4* (*TAR4; AT1G34060*) and the *Tryptophan Synthase Beta subunit* (*TSB2; AT5G28237*) homolog was specifically up-regulated in shoots. It appears that auxins are up-regulated at later stages in roots in response to a drought response relative to ABA biosynthesis. Among the cytokinin biosynthesis-related genes, the expression of *LOG2* (*AT2G35990*)*, CKX5* (*AT1G75450*), *UGT76C1* (*AT5G05870*), and *UGT73C5* (*AT2G36800*) was up-regulated specifically in roots, while the up-regulation of *LOG5* (*AT4G35190*) was delayed in shoots compared to roots (Table 2). Among the gibberellin (GK) related genes, the expression of *GA2* (*AT1G79460*) was up-regulated on days 7 and 9 of the drought stress, while the expression of *GA20ox5* (*AT1G44090*) and *GA20ox6* (*AT1G02400*) was specifically up-regulated on day 9. Among the ethylene biosynthesis-related genes, only the expression of *ACS2* (*AT1G01480*) was up-regulated in roots from day 3 to 9 of the drought stress, while the expression of other ethylene biosynthesis-related genes in roots did not change in response to drought stress (Table 2). Among the jasmonate (JA) related genes, the expression of *ACX1* (*AT4G16760*) and *ST2a* (*AT5G07010*) was up-regulated on days 7 and 9, while the expression of *ACX2* (*AT5G65110*) was up-regulated only on day 9 in roots. The majority of genes involved in brassinosteroid synthesis were down-regulated.

### Changes in the expression of transcription factors

The role of various transcription factors (TFs), such as DREB, AREB, MYC, and NAC, in regulating drought response has been previously reviewed (Yamaguchi-Shinozaki and Shinozaki, 2005, 2006; Nakashima et al., 2014). Therefore, changes in the expression of these transcription factors will not be discussed in detail. The present study focuses on TFs that were differentially up-regulated either in roots vs. shoots. The expression of eight MYB family members was specifically up-regulated in roots (Table 3). Among these, the expression of *MYB79* (*AT4G13480*) and *MYB71* (*AT3G24310*) was up-regulated on days 3–9, while *MYB20* (*AT1G66230*) was up-regulated on days 7 and 9 of the drought stress. The expression of *MYB122* (*AT1G74080*), was up-regulated only at the 3rd day of drought stress, while expression of *MYB14* (*AT2G31180*), *MYB52* (*AT1G17950*), *MYB54* (*AT1G73410*), and *MYBH* (*AT5G47390*) was up-regulated only at the 9th day of drought stress. The expression of *NAC95* (*AT5G41090*), *WRKY2* (*AT5G56270*), and *MEE8* (*AT1G25310*) was specifically up-regulated in roots on days 7 and 9. The expression of *ICE1* (*AT3G26744*) was up-regulated in roots from day 5 to 9 day of the drought stress (Table 3).

**Table 3:** **Transcription factors specifically up-regulated in roots or shoots**.

**Gene**	**Name**	**Root**				**Function**
		**3 days**	**5 days**	**7 days**	**9 days**	
*At2g31180*	*MYB14/Myb14at*	0.77	0.87	1.44	**2.43**	2R3-MYB gene family (Stracke et al., 2001)
*At1g66230*	*MYB20*	1.08	**2.23**	**6.05**	**14.34**	Negative regulator of drought stress (Gao et al., 2014)
*At1g17950*	*MYB52*	1.50	1.50	1.80	**2.54**	ABA hypersensitivity and drought tolerance (Park M. Y. et al., 2011), Cell wall biosynthesis, xylem vessel regulate lignin, xylan, and cellulose biosynthesis (Nakano et al., 2010)
*At1g73410*	*MYB54*	1.15	1.23	1.45	**2.47**	Regulate lignin, xylan, and cellulose biosynthesis (Stracke et al., 2001; Zhong et al., 2008)
*At3g24310*	*MYB71*	**5.09**	**18.70**	**28.06**	**28.59**	Starch/nectar synthesis (Liu and Thornburg, 2012)
*At4g13480*	*MYB79*	**3.44**	**34.72**	**35.08**	**25.82**	2R3-MYB gene family (Stracke et al., 2001)
*At1g74080*	*MYB122*	**2.44**	1.18	1.54	1.78	R2R3-MYB gene family (Stracke et al., 2001)
*At5g47390*	*MYBH/KUA1*	1.05	1.22	1.60	**2.17**	Controls cell expansion during leaf development by controlling ROS homeostasis. The mRNA is cell-to-cell mobile (Kwon et al., 2013)
*At2g43000*	*NAC042/JUB1*	**3.48**	1.31	0.34	0.28	H_2_O_2_ tolerance, regulates longevity (Wu et al., 2012)
*At4g01550*	*NAC69/NTM2/NTL13*	0.90	1.26	1.79	**3.98**	Seed germination under high salinity, auxin signaling (Park J. et al., 2011)
*At4g29230*	*NAC75*	1.21	1.40	1.53	**2.11**	Membrane bound (Kim S-G et al., 2010)
*At5g41090*	*NAC95*	1.19	0.94	**2.74**	**2.66**	Expresses in female gametophyte (Wang et al., 2010)
*At5g61430*	*NAC100*	**3.13**	**2.27**	1.72	**2.16**	Targeted by miR164 and involved in boundary size control (Rhoades et al., 2002; Laufs et al., 2004)
*At5g56270*	*WRKY2*	1.27	1.78	**2.24**	**2.27**	Pollen development and function (Guan et al., 2014), ABA induced germination and post-germination developmental arrest (Jiang and Yu, 2009)
*At5g46350*	*WRKY8*	1.21	1.11	1.16	**2.50**	Basal defense (Chen et al., 2010)
*At1g30650*	*WRKY14*	1.43	1.24	1.63	**2.27**	CRK2 and CRK3 phosphorylates WRKY14 (Nemoto et al., 2015)
*At2g46130*	*WRKY43*	1.37	1.13	1.34	**2.71**	Potentially interact with MAPK3 (Taj et al., 2011)
*At2g40740*	*WRKY55*	**4.47**	0.69	0.67	0.49	Potentially interact with MAPK3 (Taj et al., 2011)
*At1g66600*	*WRKY63*	1.49	1.53	1.35	**4.17**	Seedling growth, Stomatal closure, Downstream of *ABI1, ABI2, ABI3* and *ABI5*.Upstream of *ABF2, COR47*, and *RD29A* (Rushton et al., 2012)
*At3g56400*	*WRKY70*	**2.07**	0.56	0.26	0.26	ABA and GA signaling (Zhang et al., 2015)
*At1g25310*	*bHLH /MEE8*	1.09	1.53	**3.84**	**4.10**	Female gametophyte development (Pagnussat et al., 2005) Protein folding (Cho et al., 2011)
*At1g02340*	*bHLH/HFR1*	1.29	1.16	**2.01**	**4.86**	Binds to PIF1, govern light induced seed germination (Shi et al., 2013)
*At1g26945*	*PRE6/KIDARI*	1.63	1.87	**2.55**	1.64	Non-DNA binding (Hyun and Lee, 2006)
*At1g35460*	*bHLH/FBH1*	0.87	0.99	1.24	**2.02**	Induces flowering (Ito et al., 2012)
*At3g26744*	*bHLH/ICE1*	1.73	**3.33**	**4.64**	**5.75**	Upstream of DREB1B (Denay et al., 2014)
*At3g47640*	*POPEYE*	1.17	1.32	**2.02**	**2.32**	Regulates iron transport (Long et al., 2010)
*At4g29930*	*bHLH27*	1.54	**2.04**	**2.38**	1.02	Nematode susceptibility (Jin et al., 2011)
*At1g59530*	*bZIP4*	0.97	1.19	1.22	**2.08**	Potentially interact with MAPK3 (Taj et al., 2011)
		**Shoot**				
*At3g62610*	*MYB11/PFG2*	1.25	1.82	**2.68**	1.61	Phenylpropanoide pathway/Flavonol biosynthesis (Stracke et al., 2007)
*At1g06180*	*MYB13/MYBIfgn*	0.83	1.59	**10.26**	**6.36**	Abiotic stress response/Drought, light, and wounding, ABA mediated (shoot morphogenesis) (Miséra and Bäumlein, 1998)
*At3g27810*	*MYB21/MYB3*	0.99	**15.05**	**51.19**	**20.97**	Flower development, Induction by JA (Cheng et al., 2009)
*At5g40350*	*MYB24*	1.22	**4.58**	**19.73**	**9.25**	Flower specific (Cheng et al., 2009)
*At1g74650*	*MYB31/Y13*	1.19	1.48	**2.09**	0.77	
*At4g34990*	*MYB32*	1.26	1.69	**4.03**	**2.39**	Phenylpropanoide pathway (Preston et al., 2004)
*At5g06100*	*MYB33*	1.10	1.42	1.96	**2.69**	Stamen/Anther development (Millar and Gubler, 2005) Abiotic stress response/ABA sensitivity (Reyes and Chua, 2007)
*At1g16490*	*MYB58*	1.09	1.45	**2.28**	0.91	Phenylpropanoide pathway/Lignin biosynthesis (fibers and vessels (Zhou et al., 2009)
*At1g68320*	*MYB62*	1.23	1.36	**2.87**	**2.05**	Abiotic stress response/Phosphate starvation, GA mediated (Devaiah et al., 2009)
*At3g11440*	*MYB65*	1.28	1.56	1.35	**2.14**	Stamen/Anther development (Millar and Gubler, 2005)
*At5g26660*	*MYB86/MYB4*	1.82	**2.52**	**3.01**	1.62	
*At1g66390*	*MYB90/PAP2*	1.04	**6.07**	**21.85**	**8.48**	Metabolism Phenylpropanoide pathway/Anthocyanin biosynthesis (Borevitz et al., 2000)
*At5g62320*	*MYB 99/MYBUC15*	1.79	1.99	**2.05**	**4.36**	Stamen development/Anther development (tapetum) (Alves-Ferreira et al., 2007)
*At2g32460*	*MYB101/AtM1*	1.53	**2.66**	**7.91**	**16.52**	Abiotic stress response/ABA sensitivity (Reyes and Chua, 2007)
*At1g63910*	*MYB103*	0.89	1.25	**6.21**	0.84	Cell wall thickening (Zhong et al., 2008) xylem differentiation (Nakano et al., 2010)
*At3g02940*	*MYB107*	0.89	1.05	1.94	**2.29**	
*At3g55730*	*MYB109*	0.96	1.07	1.49	**2.55**	
*At5g49330*	*MYB111/PFG3*	1.05	1.51	**2.09**	0.82	Phenylpropanoide pathway/Flavonol biosynthesis (Stracke et al., 2007)
*At1g25340*	*MYB116*	1.03	1.39	**2.56**	1.48	
*At5g41020*	*MYB*	0.95	1.27	1.87	**2.33**	Potentially interact with MAPK3 (Taj et al., 2011)
*At1g70000*	*MYB*	1.28	1.55	1.92	**3.00**	Response to trehalose (Bae et al., 2005)
*At5g04410*	*NAC2/NTL11*	1.03	1.14	1.69	**2.66**	Controls organ size (Nguyen et al., 2013)
*At1g02220*	*NAC3*	0.94	0.72	0.80	**6.45**	Potentially interact with MAPK3 (Taj et al., 2011)
*At3g04410*	*NAC4*	0.89	0.91	0.88	**2.48**	Inhibition by small peptide (Seo et al., 2011)
*At1g02250*	*NAC5*	0.95	0.76	0.89	**6.63**	Potentially interact with MAPK3 (Taj et al., 2011)
*At1g32770*	*NAC12/SND1*	0.92	1.36	**14.15**	1.45	Secondary wall synthesis (Zhong et al., 2006)
*At1g33060*	*NAC014*	1.31	1.71	1.84	**2.62**	Involved in phloem parenchyma transfer cell development (Arun Chinnappa et al., 2013), Potentially interact with MAPK3 (Taj et al., 2011)
*At1g61110*	*NAC25/TAPNAC*	**2.27**	**3.76**	**4.09**	**2.76**	Apparently under the control of male sterility 1, No phenotype (Alvarado et al., 2011)
*At1G69490*	*NAC29/NAP*	0.85	**2.01**	**7.44**	**13.92**	Leaf senescence (Guo and Gan, 2006)
*At2g46770*	*NAC43/NST1*	1.30	**2.42**	**4.33**	1.20	2nd wall thickness (Mitsuda et al., 2005)
*At3g04060*	*NAC46*	1.33	**2.03**	1.45	**2.85**	Interacts with RCD1 (Jaspers et al., 2009)
*At3g04420*	*NAC48*	0.88	0.89	0.92	**6.44**	Involved in phloem parenchyma transfer cell development (Arun Chinnappa et al., 2013)
*At3g10490*	*NAC52*	1.17	1.31	1.98	**2.16**	
*At3g29035*	*NAC059/ORS1*	1.26	**2.49**	1.53	1.43	H_2_O_2_ responsive, controls senescence (Balazadeh et al., 2011)
*At3g61910*	*NAC 66/NST2*	1.29	1.34	**2.35**	0.81	2nd wall thickness (Mitsuda et al., 2005)
*At5g07680*	*NAC80*	0.73	1.38	**2.47**	**4.01**	Targeted by members of the miR164 and involved in boundary size control (Rhoades et al., 2002; Laufs et al., 2004)
*At5g14000*	*NAC84*	0.92	1.69	**2.03**	1.33	
*At5g63790*	*NAC102*	0.83	0.89	**2.13**	**2.51**	Potentially downstream of BZR1-BAM signaling pathway to control shoot growth and development (Reinhold et al., 2011)
*At1g62300*	*WRKY6*	0.97	0.95	1.07	**3.05**	Defense response (Castrillo et al., 2013)
*At2g44745*	*WRKY12*	1.15	1.71	**2.96**	1.27	Potentially interact with MAPK3 (Taj et al., 2011)
*At4g31800*	*WRKY18*	1.22	**2.56**	0.33	1.10	ABA signaling, may interact with AtWRKY40 to activate *AtWRKY60* (Rushton et al., 2012)
*At2g30250*	*WRKY25*	1.14	1.21	0.76	**2.48**	ABA sensitivity, Salt tolerance (Jiang and Deyholos, 2009)
*At3g04670*	*WRKY39*	1.00	1.01	1.37	**2.04**	Heat stress (Li et al., 2010)
*At1g80840*	*WRKY40*	0.55	**2.13**	0.60	0.79	Same as WRKY1
*At1g27660*	*bHLH protein*	1.10	1.41	**2.05**	1.58	
*At3g19500*	*bHLH protein*	1.39	1.24	1.77	**2.54**	
*At4g00050*	*bHLH /UNE10*	1.30	1.48	**2.14**	1.39	Interacts with RCD1 (Jaspers et al., 2009)
*At4g00870*	*bHLH14*	1.61	**2.15**	**2.27**	0.80	-ve regulator of JA signaling (Song et al., 2013)
*At5g48560*	*bHLH/CIB2*	1.42	1.29	**3.14**	1.95	CRY2 dependent regulation of flowering time (Liu et al., 2013)
*At5g54680*	*bHLH105/ILR3*	1.01	0.94	1.01	**2.19**	Regulates metal transport, IAA response (Rampey et al., 2006)
*At5g62610*	*bHLH*	1.15	1.22	**2.07**	**3.36**	
*At3g56980*	*bHLH39*	**4.91**	**13.19**	**12.08**	1.28	Iron related (Yuan et al., 2008; Wang et al., 2013)
*At1g25330*	*bHLH075/CESTA*	1.28	**4.06**	**7.06**	**6.89**	Positive regulator of BRs (Poppenberger et al., 2011).
*At1g10610*	*bHLH090*	1.54	**2.02**	**2.50**	**2.94**	Myrosin cell development, Defence against herbivores (Shirakawa et al., 2014)
*At2g41240*	*bHLH 100*	**3.41**	**8.38**	**7.78**	1.18	Iron transport, FIT independent (Sivitz et al., 2012)
*At1g68880*	*bZIP8*	0.87	0.84	0.86	**2.04**	Potentially interact with MAPK3 (Taj et al., 2011)
*At5g24800*	*bZIP9*	1.19	1.44	**2.78**	**4.90**	ABA induced, phloem specific (Zimmermann et al., 2004; Weltmeier et al., 2009)
*At2g41070*	*bZIP12/ DPBF4*	0.88	1.23	1.42	**2.13**	Regulate chloroplast aspirate pathway under low energy conditions (Ufaz et al., 2011)
*At3g51960*	*bZIP24*	1.07	1.01	1.01	**2.79**	Salt tolerance (Yang et al., 2009)
*At3g54620*	*bZIP25*	0.96	1.06	1.48	**3.72**	Expresses in stamen, Allocation of nutrients (Weltmeier et al., 2009)
*At3g10800*	*bZIP28*	1.04	1.05	1.10	**2.58**	Heat stress response, BR signaling (Liu et al., 2007; Gao et al., 2008; Che et al., 2010)
*At5g38800*	*bZIP43*	1.17	1.37	**3.91**	1.29	Potentially interact with MAPK3 (Taj et al., 2011)
*At1g75390*	*bZIP44*	1.43	**3.18**	**5.11**	**3.30**	Embryogenesis (Weltmeier et al., 2009)
*At2g35550*	*BPC7*	1.33	1.66	**2.86**	**3.56**	Developmental phase (Berger et al., 2011)

*Values shown in bold are significantly up or down-regulated (FDR < 0.1)*.

Various TFs were also specifically up-regulated in shoots. The expression of *NAC25/TAPNAC* (*AT1G61110*) was significantly up-regulated from day 3 to 9, while the expression of *bHLH100* (*AT2G41240*) was significantly elevated from day 3 to 7 of the drought stress (Table 3). The expression of *MYB21* (*AT3G27810*), *MYB24* (*AT5G40350*), *MYB90* (*AT1G66390*), *MYB101* (*AT2G32460*), *NAC29* (*AT1G69490*), *bHLH075*/*CESTA* (*AT1G25330*), *bHLH090* (*AT1G10610*), and *bZIP44* (*AT1G75390*) significantly increased specifically in shoots from day 5 to 9 of the drought stress.

### Changes in the expression of solute transport-related genes

The expression of genes related to the transport of amino acids and other solutes including, malate, iron (Fe), and sulfur (S) changed significantly in both roots and shoots in response to the drought stress treatment. The expression of the malate transporters *ALMT2* (*AT1G08440*)*, ALMT3* (*AT1G18420*), and *ALMT10* (*AT4G00910*) was up-regulated in roots during the early stages of the drought stress (Figure 3 and Table S1). The expression of the sucrose family transporter gene *SWEET15* (*AT5G13170*) was also significantly up-regulated in both roots and shoots (Figure 3). The expression of a MATE family member, *ZRZ* (*ZRIZI*; *AT1G58340*), which is involved in communicating a leaf-borne signal that determines the rate of organ initiation (Burko et al., 2011), was also up-regulated in roots.

Genes related to the transport of Fe, S, and other solutes were also differentially regulated in roots and shoots. Among these genes, those related to Fe transport were of particular interest. The expression of genes principally responsible for Fe uptake from the soil, i.e., *iron regulated transporter 1* (*IRT1; AT4G19690*) and *ferric reduction oxidase 2* (*FRO2*; *AT1G01580*) was significantly down-regulated in roots from day 5 to 9, indicating that plants were not uptaking Fe from soil during that time (Table S1). FRO2 reduces ferric to ferrous to increase its solubility and facilitates Fe uptake by IRT1 in plants (Jeong and Connolly, 2009). The expression of Fe transporter *IRT3* (*AT1G60960*) was also down-regulated in roots on days 7 and 9, and on day 9 in shoots. On the other hand, genes regulating Fe distribution within a plant body were significantly up-regulated during the early stages of the drought stress. The expression of nicotianamine (NA) synthase 2 (*NAS2: AT5G56080)*, which encodes a metal chelator NA, was up-regulated on day 3 and subsequently down-regulated on days 7 and 9. The expression of oligopeptide transporter 3 (*OPT3; AT4G16370*), involved in Fe distribution within a plant body (Stacey et al., 2008), was very significantly up-regulated in roots from day 3 to 7 of the drought stress. The expression of *OPT3* in shoots was down-regulated on day 9. The expression of *IRT2* (*AT4G19680*) was up-regulated in roots on day 3 and then down-regulated on days 7 and 9, while in shoots it was down-regulated on day 9 of the drought stress. The expression of a gene coding a Fe-S cluster biosynthesis family protein (*AT2G36260*) was significantly down-regulated in roots from day 5 to 9, while the expression of another gene coding an Fe-S cluster biosynthesis protein (*AT2G16710*) increased in both roots and shoots (Table S1). The expression of a mitochondrial Fe reductase, *FRO8* (*AT5G50160*) increased in roots from day 5 to 9. The expression of the metal transporters *YSL2* (*AT5G24380*) and *VIT1* (*AT2G01770*) increased in roots on day 7 of the drought stress (Table S1), while the expression of *FRO4* (*AT5G23980*) decreased in roots and increased in shoots (Tables S2, S3). Many bHLH transcription factors reported to be involved in Fe homeostasis were differentially regulated in roots and shoots. The expression of the transcription factors regulating Fe uptake/translocation *POPEYE* (*AT3G47640*) and *BRUTUS* (*BTS; AT3G18290*) was up-regulated in roots on days 7 and 9 (Table 3 and Table S1), while the expression of *bHLH115* (*AT1G51070*) was down-regulated in roots on days 7 and 9 (Table S2). The expression of *bHLH38* (*AT3G56970*) increased in roots and shoots from day 5 to 7, while that of *bHLH39* (*AT3G56980*) increased specifically in shoots from day 3 to 7 of the drought stress. The expression of *bHLH101* (*AT5G04150*) increased in roots on day 3, and from day 5 to 9 in shoots. Collectively, these results suggest that Fe distribution within a plant body can significantly change during the course of a drought stress.

### Changes in the expression of genes related to transcription regulation and chromatin synthesis/modification

In *Arabidopsis*, genes controlling epigenetic changes that occur in response to abiotic stresses have been reported (Kim et al., 2015). In the present study, we focused on the differential expression of genes related to chromatin structure or chromatin modification in both roots and shoots. The expression of *AtRRP6L1* (*AT1G54440*), which controls DNA methylation; *Early Flowering 8* (*ELF8; AT2G06210*), which is putatively involved in regulating gene expression; and *Demeter Like 1* (*DML1*; *AT2G36490*), a repressor of transcriptional silencing; was significantly up-regulated in roots on day 9 of the drought stress. The expression of *AGO4* (*AT2G27040*), which is involved in siRNA-mediated gene silencing, was up-regulated in roots on day 7; and *DRM2* (*AT5G14620;* methyl transferase) was up-regulated on days 7 and 9 of the drought stress (Table 4). The expression of *Histone DeAcetylase 8* (*HDA8; AT1G08460*) was up-regulated in both roots and shoots on day 9. Changes in the expression of various histone protein-related genes were also observed. The expression of histone *H1-3* (*AT2G18050*) was significantly up-regulated in roots from day 5 to 9, and from day 3 to 9 in shoots. On the other hand, the up-regulation of *HTR6/H3.6* (*AT1G13370*) and *HTR14/H3*.14 (*AT1G75600*) occurred earlier in roots than in shoots. The expression of *HTR10/H3.10* (*AT1G19890*) was specifically up-regulated in shoots (Table 4). Many histone-related genes were also significantly down-regulated in both roots and shoots (Table 4), indicating that chromatin structure changes significantly in plants under drought stress conditions.

**Table 4:** **Changes in expression of chromatin related genes**.

**Gene**	**Name**	**Roots**				**Shoots**			
		**3 days**	**5 days**	**7 days**	**9 days**	**3 days**	**5 days**	**7 days**	**9 days**
*At1g54440*	*RRP6L1*	0.72	0.90	1.33	**2.37**	1.04	1.13	1.28	1.30
*At2g27040*	*AGO4*	1.06	1.64	**2.05**	1.70	1.09	1.27	1.30	0.59
*At5g14620*	*DRM2*	0.84	1.15	**2.02**	**3.33**	1.01	1.01	1.30	1.55
*At2g06210*	*ELF8*	0.83	1.19	1.54	**2.46**	1.11	1.17	1.10	1.17
*At2g36490*	*DML1/ATROS1*	0.93	1.39	1.95	**2.47**	1.30	1.50	1.57	1.01
*At1g79000*	*HAC1*	1.16	1.77	**2.99**	**3.38**	0.99	1.03	1.23	**2.11**
*At1g08460*	*HDA8*	1.30	1.50	1.71	**2.32**	1.32	1.37	1.73	**2.39**
*At1g21920*	*Histone H3K4 methyltransferase*	0.94	0.85	1.11	1.60	1.15	1.37	1.49	**2.28**
*At1g77300*	*ASSH2*	1.02	1.08	**2.01**	1.82	**2.18**	1.00	0.94	1.25
*At2g44950*	*HUB1/RDO4*	0.79	0.95	1.33	**2.44**	1.10	1.29	1.46	**2.21**
*At1g55250*	*Histone mono-ubiquitination 2*	0.89	0.91	1.15	1.44	1.27	1.55	1.72	**2.43**
*At2g18050*	*Histone H1-3*	1.31	**5.61**	**17.82**	**19.15**	**4.13**	**18.46**	**60.70**	**57.35**
*At5g02560*	*Histone H2A 12*	1.26	1.01	0.90	0.50	1.50	1.79	**2.29**	1.46
*At5g27670*	*Histone H2A 7*	0.99	1.00	1.50	1.38	1.16	1.08	**2.12**	1.54
*At1g13370*	*HTR6/H3.6*	1.53	**4.41**	**8.81**	**22.32**	1.11	0.91	**2.74**	**30.28**
*At1g75600*	*HTR14/H3.14*	1.86	**6.23**	**13.74**	**32.84**	0.96	0.93	**5.70**	**85.41**
*At1g19890*	*HTR10/H3.10*	1.15	1.12	0.94	1.02	1.02	1.41	**2.03**	**2.69**
*At5g02570*	*HTB10/H2B.10*	1.25	1.20	1.69	**2.11**	1.07	1.04	1.81	**2.65**
*At1g51060*	*Histone H2A 10*	1.08	0.89	0.51	**0.26**	1.00	0.82	0.51	**0.31**
*At5g65350*	*Histone 3 11*	**0.45**	**0.50**	**0.40**	**0.45**	0.57	0.77	0.60	**0.49**
*At2g28720*	*Histone superfamilyHTB3/H2B.3*	0.86	**0.49**	**0.35**	**0.24**	0.99	1.18	0.78	**0.40**
*At3G45980*	*Histone superfamily/H2B*	0.85	0.72	**0.49**	**0.23**	1.07	0.89	0.67	**0.20**
*At3g53650*	*Histone superfamily/H2B*	1.01	0.89	0.59	**0.31**	0.86	0.58	**0.44**	0.68
*At5g12910*	*Histone superfamily/H3.3*	1.05	0.95	**0.50**	**0.13**	0.85	0.55	**0.39**	**0.09**
*At5g10390*	*Histone superfamily/H3*	1.07	1.14	0.58	**0.14**	0.83	0.56	**0.44**	**0.08**
*At5g10400*	*Histone superfamily/H3*	0.83	0.80	0.56	**0.20**	0.76	0.59	**0.40**	**0.10**
*At5g65360*	*Histone superfamily/H3.1*	1.09	1.21	0.91	**0.26**	0.78	**0.42**	**0.31**	**0.08**
*At3g46320*	*Histone superfamily /H4/HFO1*	0.99	0.98	0.71	**0.25**	0.80	0.52	**0.45**	**0.12**
*At5g59690*	*Histone superfamily /H4/HFO2*	0.94	0.87	0.68	**0.33**	0.81	0.53	**0.49**	**0.22**
*At3g53730*	*Histone superfamily/HFO5*	0.86	0.86	0.77	**0.47**	1.07	1.01	0.92	**0.49**
*At5g59970*	*Histone superfamily/H4/HFO6*	0.98	0.98	0.87	**0.33**	0.86	0.64	**0.44**	**0.18**
*At1g01370*	*Histone superfamily/HTR12*	0.86	0.89	0.68	**0.41**	0.94	0.78	0.56	**0.32**
*At3g45930*	*Histone superfamily/H4/HFO7*	0.95	0.98	0.76	**0.32**	0.80	0.61	**0.49**	**0.18**

*Values shown in bold are significantly up or down-regulated (FDR < 0.1)*.

## Discussion

Transcriptomic changes in *Arabidopsis* in response to drought stress have been previously reported (Kreps et al., 2002; Seki et al., 2002, 2007; Shinozaki et al., 2003; Huang et al., 2008; Matsui et al., 2008). Global changes of gene expression from both roots and shoots of drought-stressed *Arabidopsis* plants under soil conditions, however, has not been investigated. Thus, our data provide new information pertaining to the differential regulation of genes in shoots vs. roots in response to drought stress. It should be noted that plants started flowering during drought stress (Figure S1), thus numerous genes and transcription factors related to flowering were also up-regulated in shoots. This could be a potential interference in understanding the drought responsive genes, particularly in shoot tissue.

### Root to shoot signaling during a drought stress

The differential regulation of ABA biosynthesis- and transport-related genes highlights the importance of root to shoot signaling in response to drought stress. NCEDs are considered to be limiting factors in ABA synthesis and signaling, and the suppression of *NCED3* results in severe sensitivity to drought (Iuchi et al., 2001). The expression of *NCED3* has been reported to be up-regulated in both roots and shoots in response to drought stress (Behnam et al., 2013). In the current study, the expression of *NCED5* and *NCED9* was specifically up-regulated in roots. Additionally, the induction of *NCED2* in roots occurred earlier in the response to drought stress than it did in shoots (Table 1). The *Arabidopsis* genes involved in ABA transport have also been characterized. ABCG25 is a drought- and ABA-inducible plasma membrane protein that exports ABA from the vascular system (Kuromori et al., 2010). Our data indicated that expression of *ABCG25* was up-regulated in both roots and shoots by the drought stress. ABCG40 is a plasma membrane ABA influx transporter, which is highly expressed in guard cells (Kang et al., 2010). *ABCG40* knockout mutants (*atabcg40*) exhibit defects in stomatal closure in response to osmotic stress and application of ABA (Kang et al., 2010). In the current data, the expression of *ABCG40* was significantly down-regulated in both roots and shoots in response to drought stress (Table 1). The down-regulation of *ABCG40* in roots was observed on days 7 and 9 of the drought stress, whereas it was down-regulated from day 5 to 9 in shoots (Table 2).

AIT1, a member of the nitrate transporter gene family, also transports ABA (Kanno et al., 2012) and its expression was differentially regulated in roots and shoots in the current study (Table 1). Our data indicates that *AIT1, ABCG25*, and *ABCG40* are differentially regulated in response to drought stress, thus it will be important to determine if additional ABA transporters are involved in ABA transport in response to drought stress. *Arabidopsis* ABA-deficient mutants are more sensitive to drought stress than *abcg25* and *abcg40* mutants, suggesting that additional transporters with redundant functions may also be involved in ABA transport (Osakabe et al., 2014). Passive ABA transport may also contribute to signaling (Seo and Koshiba, 2011). The specific up-regulation of *NCED5* and *NCED9* in roots, as well as the earlier induction of *NCED2* in roots than in shoots, indicate that ABA signaling may originate in roots. It has been suggested that the root to shoot transport of ABA is not required since ABA produced in leaves effectively triggers ABA signaling and stomatal closure (Christmann et al., 2007). The differential up-regulation of genes involved in ABA synthesis and transport in roots vs. shoots suggest that ABA may be transported from roots to shoots. The specific up-regulation of *ABCG22* in roots further supports this idea. Although the substrate of ABCG22 has not been determined, ABCG22 has been reported to be involved in the regulation of stomata, and knock down mutants of *ABCG22* (*atabcg22*) exhibit lower leaf temperature and are drought sensitive (Kuromori et al., 2011). While *ABCG22* has been reported to be expressed in aerial organs (Kuromori et al., 2011), the specific up-regulation of *ABCG22* in roots in response to a drought stress in the current study suggests that it may also be involved in root to shoot signaling to control stomatal closure.

### Transcription factors differentially regulate the transcriptome in roots and shoots

Changes in the expression of various transcription factors were observed in roots and shoots. As plants were flowering during drought stress, the expression of various TF putatively involved in response to flowering was also up-regulated in shoots (Table 3). The role of MYB family transcription factors in controlling primary and secondary metabolism, development, cell fate and identity, and responses to different biotic and abiotic stresses has been reported (Lippold et al., 2009; Zhou et al., 2009; Dubos et al., 2010; Nakano et al., 2010; Park M. Y. et al., 2011; Katiyar et al., 2012; Liu and Thornburg, 2012; Wang and Dixon, 2012; Arun Chinnappa et al., 2013; Kwon et al., 2013; Gao et al., 2014; Kosma et al., 2014; Baldoni et al., 2015). In addition to being involved in floral development, MYB transcription factors also play a significant role in plant adaptation to drought stress, including the regulation of stomatal movement, and the induction of suberin synthesis in cuticles (Lippold et al., 2009; Park M. Y. et al., 2011; Gao et al., 2014; Baldoni et al., 2015). Synthesis of lignin and suberin in plants is controlled by VND6 and SND1, and the involvement of many MYB proteins in this process has been previously reported (Ohashi-Ito et al., 2010). MYB58 and MYB63 are known to regulate the lignin biosynthetic pathway (Zhou et al., 2009), while MYB52, MYB54, MYB85, MYB42, MYB43, MYB69, and MYB20 have been suggested to be involved in the regulation of secondary cell wall synthesis (Zhong et al., 2008). In the current study, the expression of *MYB61*, which influences lignin deposition (Newman et al., 2004), was up-regulated in both roots and shoots from day 5 to 7 of the drought stress. MYB41 has been recognized as a key regulator in cell wall expansion and modification under stress conditions (Lippold et al., 2009; Kosma et al., 2014). The up-regulation of MYB41 was observed in roots on day 7 and 9 and on day 9 in shoots.

Since various genes putatively involved in lignin and suberin biosynthesis, and secondary wall modifications were up-regulated in roots from day 3 to 9 of the drought stress; it seems reasonable that other MYB proteins may also be controlling the lignin/suberin biosynthesis in roots. The expression of *MYB20* was specifically up-regulated in roots on days 7 and 9 of the drought stress. ABA-dependent stomatal closure is impaired in plants over expressing *MYB20*, resulting in an increased susceptibility to drought stress. An opposite phenotype is associated with a *MYB20* knockout mutation, indicating that MYB20 may act as a negative regulator of ABA-mediated stomatal closure (Gao et al., 2014). It is plausible that specific up-regulation of *MYB20* in roots may be involved in ABA sensing or signaling. The protein bHLH122 plays an important role in drought and osmotic stress tolerance in *Arabidopsis* and in the repression of ABA catabolism. bHLH122 can bind directly to G-box/E-box cis-elements in the *CYP707A3* promoter and repress its expression. Furthermore, up-regulation of *bHLH122* substantially increases cellular ABA levels (Liu et al., 2014). The expression of *bHLH122* was up-regulated in roots from day 3 to 9 of the drought stress and from day 5 to 9 in shoots. Importantly, the suppression of *CYP707A3* was also observed in roots and shoots (Table 1). We suggest that the differential regulation of MYB (particularly *MYB71 and MYB79*), bHLHs (such as *ICE1, bHLH27, bHLH075, bHLH090, bHLH100*), *WRKY*, and *NAC* transcription factors in both roots and shoots (Table 3), indicate that these transcription factors may differentially regulate root and shoot response to a drought stress.

### Genes related to osmoprotectant synthesis and solute transport are differentially regulated in roots and shoots in response to a drought stress

The expression of a variety of genes involved in the synthesis of proline, galactinol, and raffinose were differentially expressed in roots and shoots in response to the drought stress. Our data confirms that the expression of *GolS1* and *GolS2* is up-regulated by drought stress (Taji et al., 2002). In addition, our microarray analysis revealed that the expression of *GolS4* was significantly up-regulated in roots from day 3 to 9 (Table 1). Differential regulation of genes involved in proline synthesis in roots and shoots was also observed in the current study. The expression of *P5CS1* was up-regulated in roots and shoots from day 5 to 9 of the drought stress (Table S1), while the expression of *P5CS2* was specifically up-regulated in shoots on days 7 and 9 (Table S3).

Malate and mannitol concentrations change in response to a water deficit and have been suggested to play a prominent role in osmotic adjustment in response to a water deficit (Lance and Rustin, 1984; Popp and Polania, 1989; Tarczynski et al., 1993; Martinoia and Rentsch, 1994; Tschaplinski and Tuskan, 1994; Karakas et al., 1997). Our results indicate that expression levels of various malate transporters, *MATE* family efflux transporters and *MSF* transporters were significantly up-regulated in roots (Table S1). The MATE and MSF family members transport a diverse range of substrates. These results indicate that during early drought stress, several transporters putatively involved in malate, amino acids, and ion transport are up-regulated and that the up-regulation of these transporters in roots could contribute to osmotic adjustments and stress signaling. The importance of differential regulation of S metabolism under drought stress has been well recognized (Chan et al., 2013), however, changes in Fe metabolism in response to drought stress has not been extensively discussed. Iron deficiency triggers a complex set of reactions in plants in order to increase Fe uptake from the soil, including developmental and physiological changes. Over the past decade, many transporters in *Arabidopsis* involved in the absorption and distribution of Fe have been identified (Conte and Walker, 2011). The transcription factor FIT1 (bHLH029) controls the expression of the Fe uptake machinery genes *FRO2* and *IRT1* in roots. In the current study, the expression of *FIT1* was down-regulated in roots from day 5 to 9 of the drought stress and on day 9 in shoots. Similarly, the expression of *FRO2, IRT1*, and *IRT3* was also down-regulated. In contrast, the expression of *bHLH38, bHLH39, bHLH100*, and *bHLH101* was significantly up-regulated. bHLH038 and bHLH039 interact with FIT, while bHLH100 and bHLH101 do not regulate FIT target genes and are reported to play a crucial role in the distribution of Fe within a plant (Yuan et al., 2008; Sivitz et al., 2012; Kobayashi et al., 2014). BTS is a negative regulator of Fe deficiency response and interacts with bHLH104, ILR3, and bHLH115 (Long et al., 2010). Down-regulation of FIT-dependent response and up-regulation of *NAS2, OPT3, IRT2, YSL2, and FRO8* suggest that the distribution of Fe within plant/cell changes significantly in response to a drought stress. There is increasing evidence that the genes involved in Fe deficiency response in plants are regulated by different plant hormones such as ABA, auxin, ethylene, GK and JA (Kobayashi et al., 2014). ABA improves Fe utilization by increasing root to shoot translocation of Fe under Fe deficiency (Lei et al., 2014). It would be interesting to investigate if the root to shoot translocation of Fe and ABA is synchronized under drought stress.

Changes in the availability of Fe significantly alters plants metabolism and could trigger localized signals (Bashir et al., 2011; Vigani et al., 2013b, 2016). Our data indicate that the expression of several 2OG-Fe(II) oxygenases was up-regulated in roots during the early stages of the drought stress. In plants, 2OG-Fe(II) oxygenases are involved in the synthesis of phytosiderophores (Nakanishi et al., 2000) and numerous other biosynthetic pathways. It was recently suggested that plant 2OG-Fe(II) oxygenases may play a role in Fe sensing and metabolic reprogramming in response to Fe-deficient conditions (Vigani et al., 2013a; Bashir et al., 2014). The up-regulation of different 2OG dioxygenases in roots observed in the current study suggests that these genes may also be involved in signaling under drought stress conditions.

### Changes in the expression of genes related to chromatin synthesis/modification

Transcriptional and post-transcriptional regulation of RNA facilitates the adjustment of plants to various abiotic stresses. Small RNAs, alternative splicing, and RNA-binding proteins are known to regulate plant stress responses (Nakaminami et al., 2012). Differential changes in the expression of various genes related to these mechanisms were observed in roots and shoots (Table 4). Modifications in chromatin structure could also significantly alter gene expression in plants responding to different abiotic stresses (Chinnusamy and Zhu, 2009; Kim J-M et al., 2010; Kim et al., 2012, 2015). The differential expression of genes involved in RNA regulation, histone modification, and several other histone-related genes observed in the current study (Table 4) indicates that epigenetic responses to a drought stress may also be differentially controlled in roots and shoots. Moreover, in addition to genes involved in acetylation, methylation, and demethylation; changes in the expression of genes encoding different histone proteins could also contribute to transcriptional changes that occur in response to a drought stress.

## Conclusions

Current studies indicate that a large number of genes belonging to diverse functional groups are differentially regulated in roots and shoots in response to a progressive drought stress. Thus, dissecting the root and shoot transcriptome may provide novel insights to understand the regulation of genes in response to different abiotic stresses. Transcriptional changes during early drought stress in roots were of particular interest. Genes involved in ABA synthesis, ABA and solute transport were up-regulated during early drought stress in roots. Various members of MFS transporters family, MATE efflux transporters, microRNA genes, suberin, pectin and secondary cell wall biosynthesis/modification-related genes, pre-tRNA genes, and various S-adenosyl-L-methionine (SAM) dependent transferases were also significantly up-regulated in roots. Moreover, our data also revealed the differential regulation of several genes involved in drought stress response and chromatin changes. The identification of genes that are highly responsive at the early stages of a drought stress, and that are differentially regulated in roots and shoots, significantly advances our understanding about early drought stress response in roots and shoots. These results can serve as an aid in the selection of root- and shoot-specific genes/promoters that could be utilized to potentially develop drought tolerant plants through molecular breeding.

## Author contributions

SR, KB, AM, and MS designed the study, SR, KB, and MT performed the research. AM, SR, and KB, analyzed the data, SR, KB, AM, and MS discussed the data and wrote the manuscript.

### Conflict of interest statement

The authors declare that the research was conducted in the absence of any commercial or financial relationships that could be construed as a potential conflict of interest.
