# Supplementary material for: Transcriptomic Analysis of Soil-Grown Arabidopsis thaliana Roots and Shoots in Response to a Drought Stress
Source: Front Plant Sci. 2016 Feb 23;7:180. doi: 10.3389/fpls.2016.00180 (PMC4763085; doi:10.3389/fpls.2016.00180)
Supplement: Supplementary file 7 [file Presentation1.pdf]

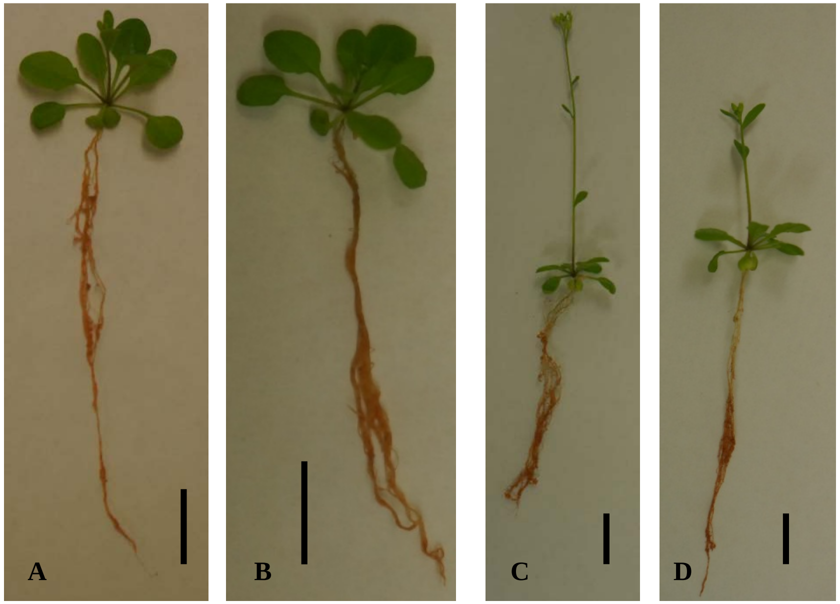

Figure S1: ***Arabidopsis*** **plants during a progressive drought stress.**
(**A-B**): Plants collected at day 0 of drought treatment. (**C-D**): Plants collected at day 5 of drought treatment. Scale bar = 1cm

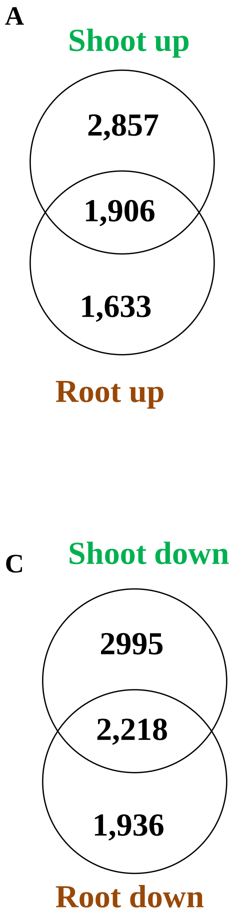

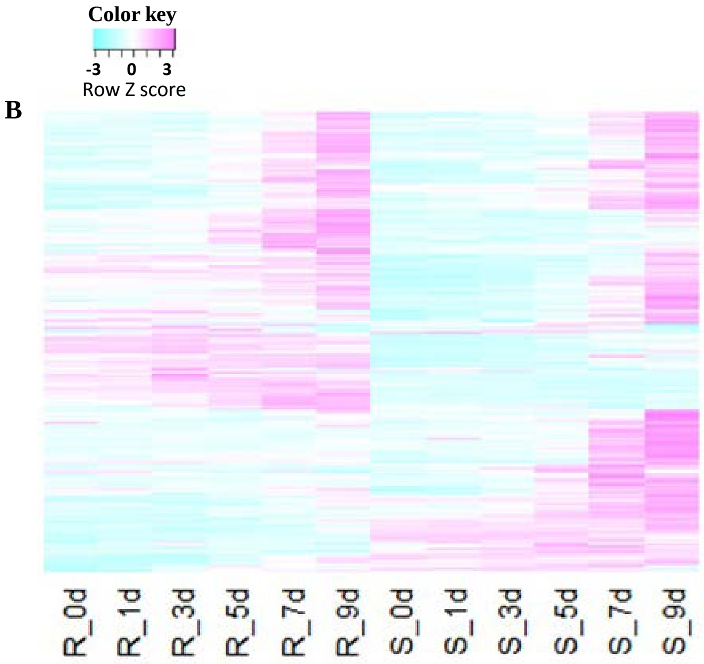

D

	1d	3d	5d	7d	9d
Shoot	0	292	899	2327	3742
Root	0	91	528	2442	4848

Figure S2: Summary of changes in gene expression in roots and shoots of *Arabidopsis* plants in response to a progressive drought stress. (**A**). Venn diagram indicating the number of genes up-regulated in roots and shoots in at least one stage of the progressive drought stress. (**B**). Heat map illustrating changes in gene expression in roots and shoots during different stages of the progressive drought stress. (**C**). Venn diagram indicating the number of genes significantly down-regulated in roots and shoots in at least one stage of the progressive drought stress. (**D**). Number of genes significantly down-regulated at different stages of the progressive drought stress.

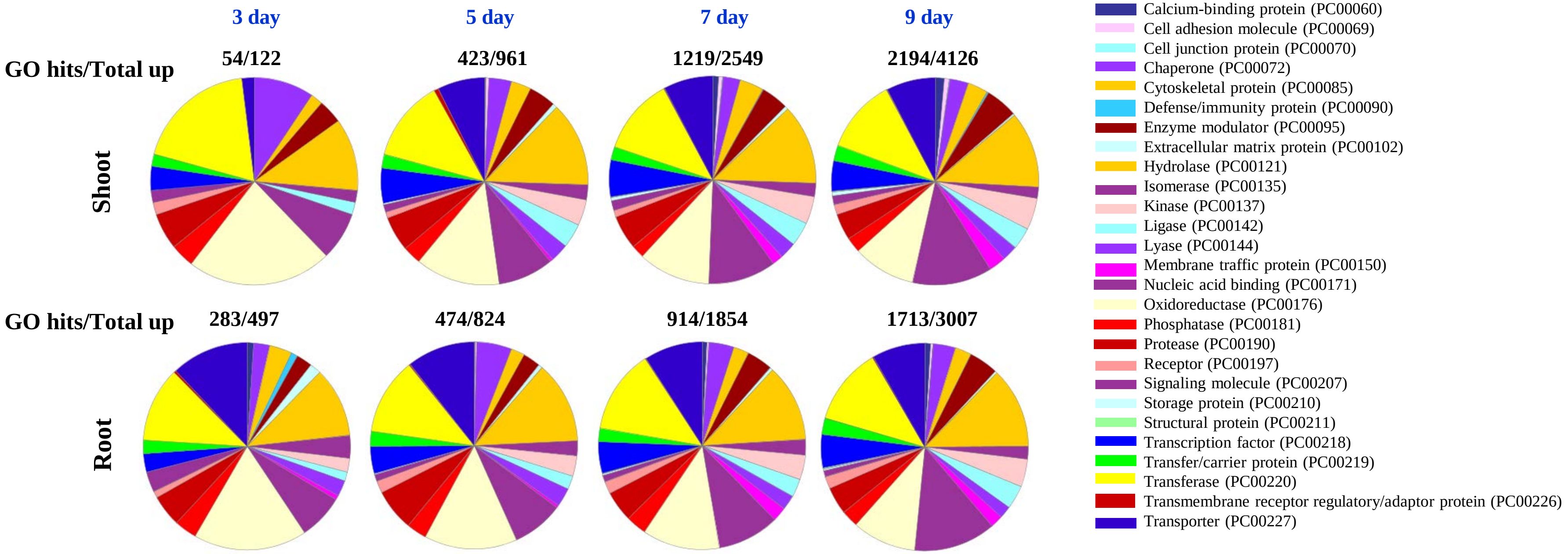

Figure S3: GO enrichment analysis (protein classification) of genes in roots and shoots of *Arabidopsis* plants in response to a progressive drought stress.

Go functional analysis for protein classification was performed using the panther classification system maintained at http://pantherdb.org/

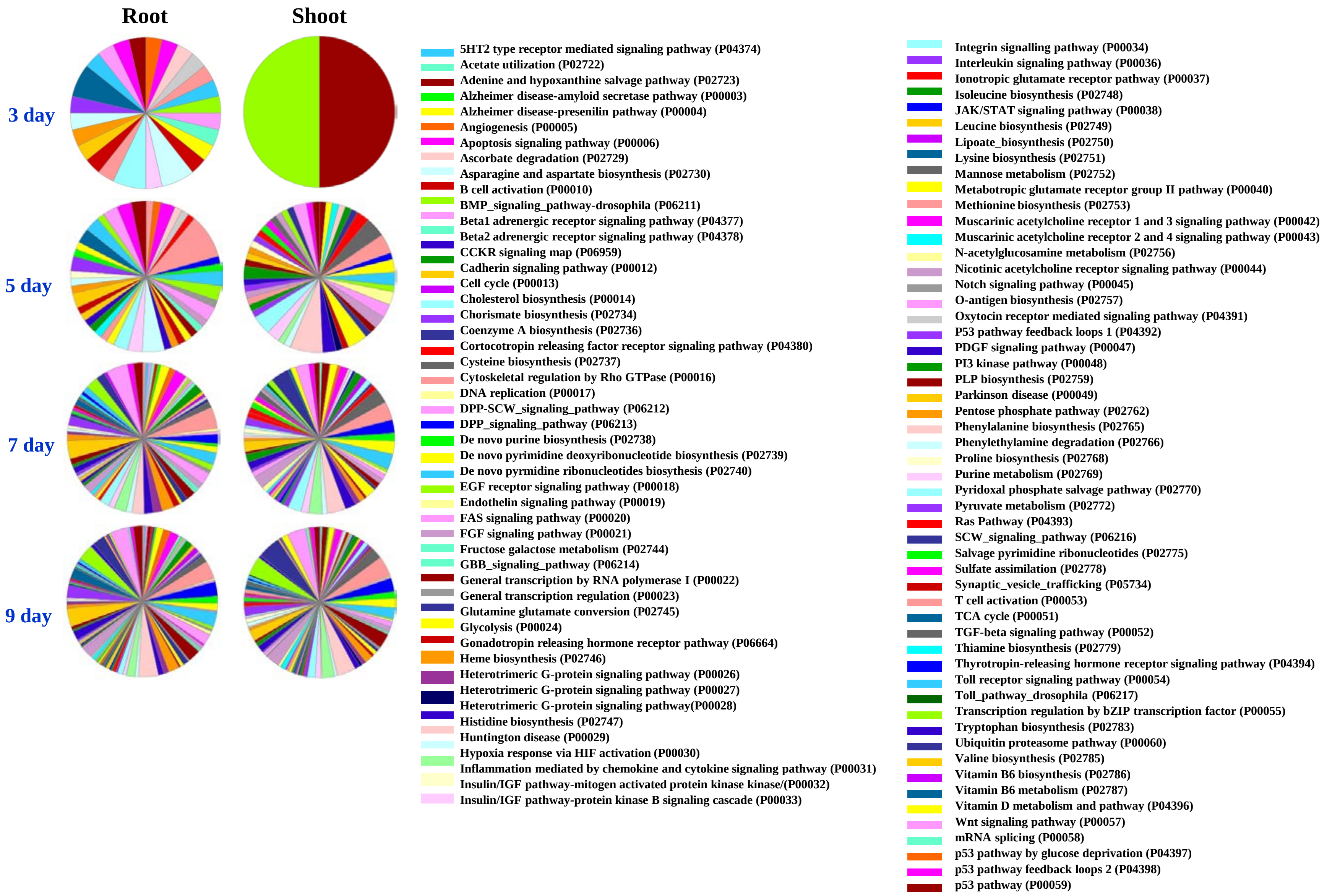

Figure S4: GO enrichment analysis (pathway) of genes in roots and shoots of *Arabidopsis* plants in response to a progressive drought stress.

Go functional classification for pathway was performed using the panther classification system maintained at http://pantherdb.org/.

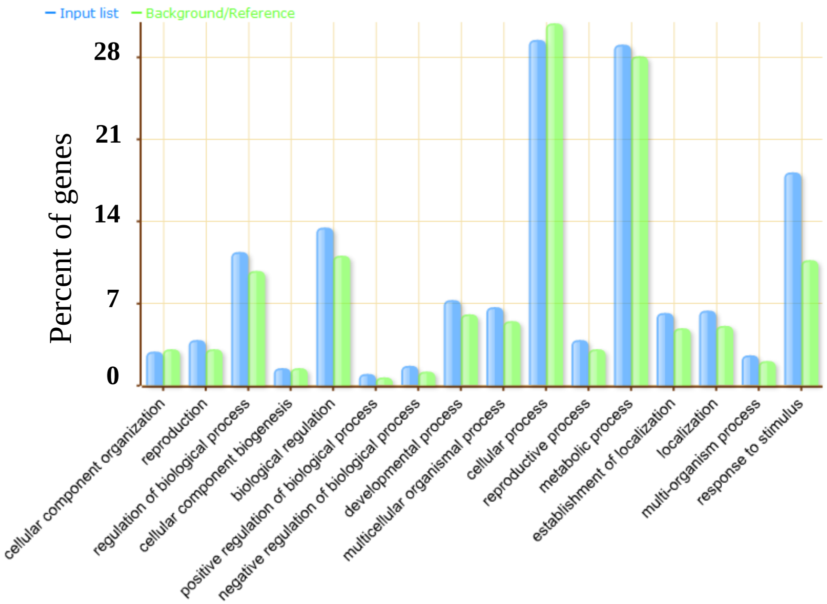

Genes upregulated in roots and shoots

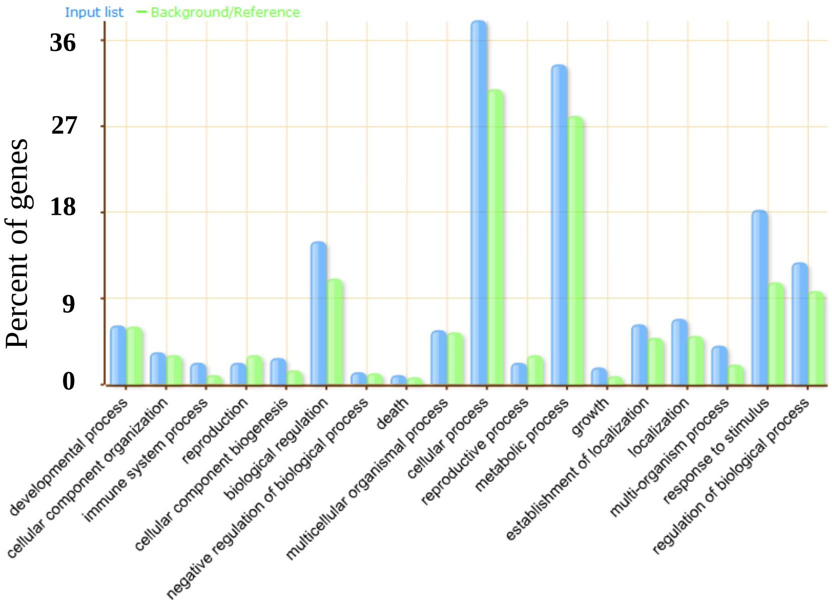

Genes down regulated in roots and shoots

Figure S5: Summary of GO enrichment analysis for genes up or down regulated both in roots and shoots of *Arabidopsis* plants in response to a progressive drought stress.

The GO analysis were performed by AGRiGO maintained at http://bioinfo.cau.edu.cn/agriGO/

Blue columns show input list, while green columns show background/reference.

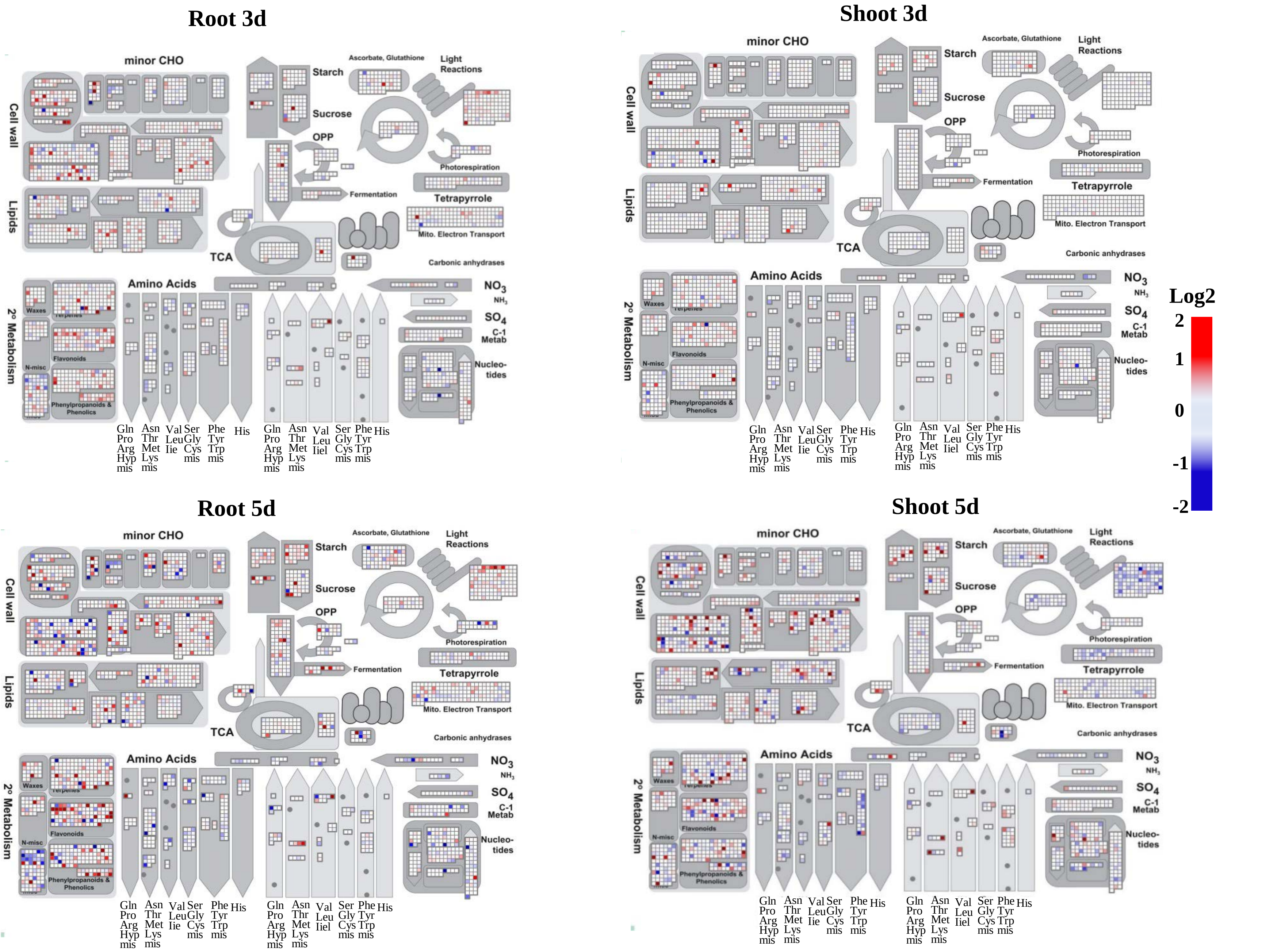

FigureS6: Differential expression of genes in roots and shoots of *Arabidopsis* at day 3 and day 5 of a progressive drought stress (MapMan 3.6.0RC1). Genes up-regulated by drought stress are shown as red, while the genes down-regulated are shown as blue.

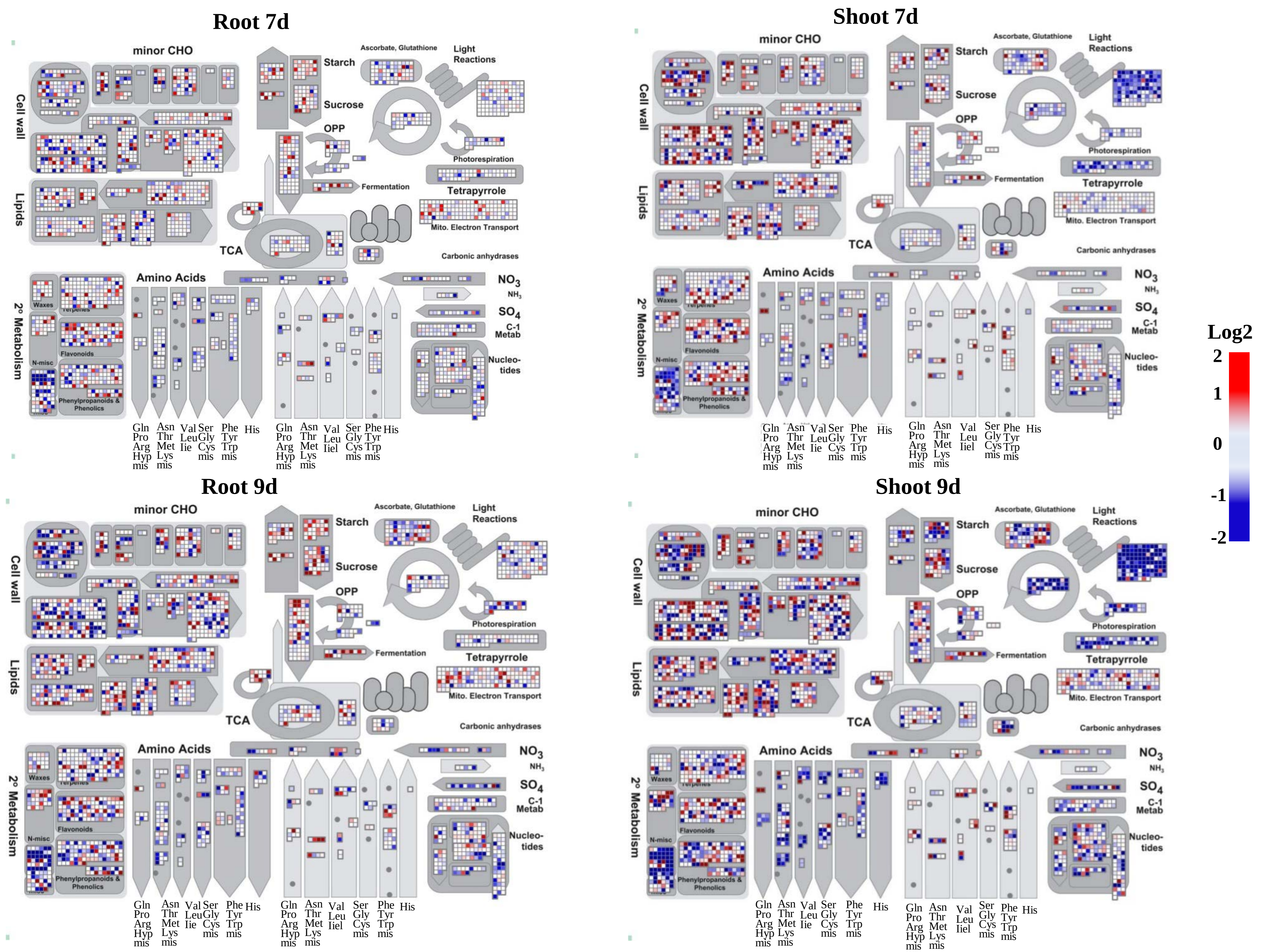

FigureS7: Differential expression of genes in roots and shoots of *Arabidopsis* at day 7 and day 9 of a progressive drought stress (MapMan 3.6.0RC1). Genes up-regulated by drought stress are shown as red, while the genes down-regulated are shown as blue.

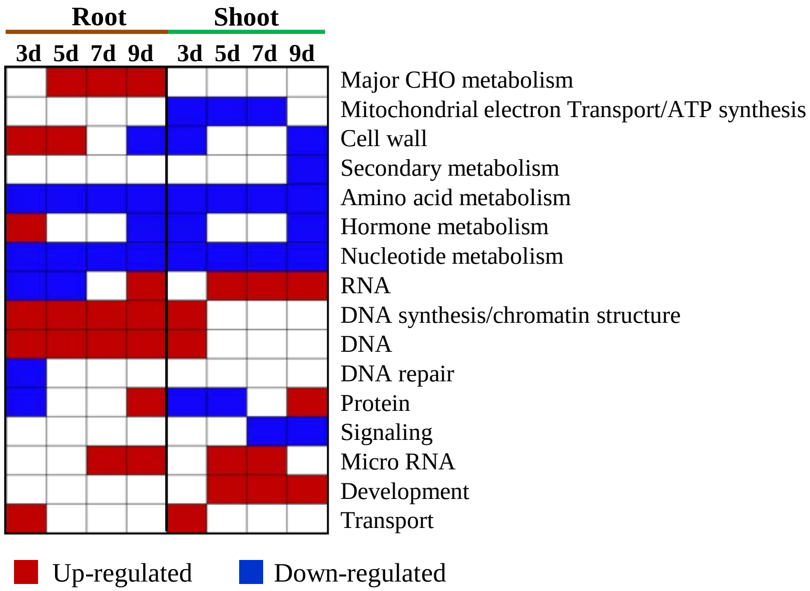

Figure S8: Summary of PageMan analysis of up- and down-regulated bins in roots and shoots of *Arabidopsis* plants during a progressive drought stress.
The bins shown in red are significantly up-regulated, while bins shown in blue are significantly down-regulated according to PageMan analysis (Wilcoxon test with BH correction, MapMan 3.6.0RC1).
